# Ethnopharmacology, phytochemistry, and pharmacology of sea buckthorn (*Hippophae rhamnoides* L.): a comprehensive review

**DOI:** 10.3389/fphar.2026.1759697

**Published:** 2026-03-06

**Authors:** Mingqi Liu, Tiantian Yu, Ulipan Nurlan, Zeyu Wu, Jin Zhao

**Affiliations:** 1 Department of Cariology and Endodontics, The First Affiliated Hospital of Xinjiang Medical University (The Affiliated Stomatology Hospital of Xinjiang Medical University), Urumqi, China; 2 Stomatology Disease Institute of Xinjiang Uyghur Autonomous Region, Xinjiang Medical University, Urumqi, China

**Keywords:** ethnopharmacology, *Hippophae rhamnoides*, pharmacology, phytochemistry, sea buckthorn

## Abstract

This review provides a comprehensive synthesis of the ethnopharmacological uses, modern applications, phytochemistry, and pharmacological mechanisms of *Hippophae rhamnoides L*. (*H. rhamnoides*) It begins by detailing its foundational role in traditional medical systems within its native range, including Tibetan, Mongolian, and Chinese medicine, as well as its broader Eurasian ethnobotanical applications. The work then systematically outlines the plant’s diverse modern utilizations in nutraceuticals, cosmeceuticals, pharmaceuticals, and environmental remediation. A thorough organ-specific analysis of its phytochemical architecture that identifies key bioactive constituents in berries, seeds and leaves links to demonstrated pharmacological effects, including hepatoprotective, anti-inflammatory, cardioprotective and neuroprotective activities. A critical discussion on the potential interference of Pan-Assay Interference Compounds (PAINS) is included to provide a necessary caveat for interpreting bioactivity data. Finally, the review identifies persistent challenges including phytochemical standardization and the translational gap between preclinical and clinical research, and proposes future research directions focused on rigorous clinical trials, mechanistic studies and sustainable exploitation within a circular bioeconomy framework.

## 1 Introduction

Global interest in phytotherapeutic interventions is increasing across both developed and developing economies, where botanical preparations are utilized not only as therapeutic adjuvants but also as prophylactic agents for health maintenance (Serafini et al., 2012). A recent review by Dubey et al. underscores the potential of *H. rhamnoides* as a multifaceted dietary supplement, highlighting its rich phytochemical profile and diverse therapeutic applications that align with its historical ethnopharmacological uses (Dubey et al., 2024). Belonging to the Elaeagnaceae family, *H. rhamnoides* is a deciduous shrub or small tree with a circumboreal distribution. Its range encompasses significant populations in the alpine ecosystems of the Indian Himalayas, Palearctic regions (e.g., China, Russia), and the boreal zones of North America and Europe (Mihal et al., 2023). In China, *H. rhamnoides* is distributed across more than 10 provinces, spanning diverse biomes from the xeric northwest to the temperate north, continental northeast, and montane southwest (Wang et al., 2012). The earliest documented ethnopharmacological applications of *H. rhamnoides* originate from China, as recorded in historical materia medica and codified in the Chinese Pharmacopoeia. The pharmacopoeia specifies its use to alleviate bronchial hypersecretion, improve dyspepsia, and enhance splanchnic circulation, with indications including mucopurulent expectoration, functional dyspepsia, alimentary-derived visceral pain, hematogenic amenorrhea, and contusion-induced ecchymosis. Ethnopharmacological records further corroborate its multifunctional role across Asia, Fennoscandia, and the Baltic territories, where it has been utilized in nutrition, bioenergy, ethnomedicine, zootherapy, the fabrication of agricultural implements, and the establishment of living barrier systems (Suryakumar and Gupta, 2011).

Over the past 3 decades, the nutraceutical and pharmacotherapeutic potential of *H. rhamnoides* has attracted growing scientific interest. The term “nutraceutical,” a blend of “nutrition” and “pharmaceutical,” was originally proposed by Stephen DeFelice in 1989 to describe “food or food components that confer medical or health benefits, including disease prevention and/or treatment” (Santini et al., 2018). This concept covers diverse bioactive-rich products, ranging from isolated nutrients and herbal supplements to functional foods, which aligns with the multifunctional characteristics of *H. rhamnoides* (Souyoul et al., 2018; Ashrafpour and Ashrafpour, 2025). Importantly, while “nutraceutical” is widely used in academic and industrial contexts, it lacks legal recognition as a distinct category for herbal products or dietary supplements under international regulatory systems(Thakur et al., 2024; Chatterjee et al., 2024). For instance, the U.S. Food and Drug Administration (FDA) and European Food Safety Authority (EFSA) classify such products as “food supplements” or “functional foods” instead of “nutraceuticals,” and despite efforts to standardize the term, no globally uniform definition has been adopted (Santini et al., 2018; Tomar et al., 2023).

**FIGURE 1:**
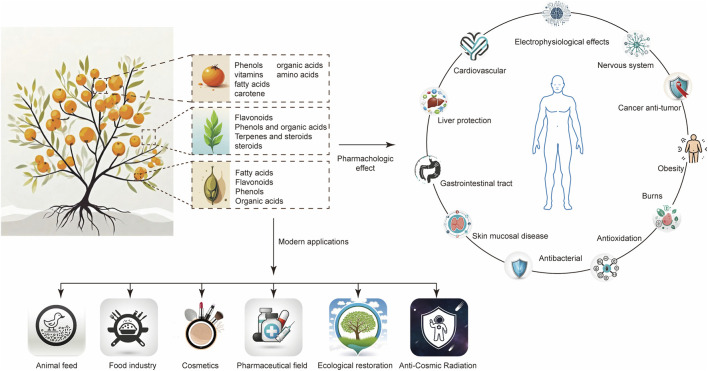
An integrative schematic of *Hippophae rhamnoides* L.: phytochemical constituents, utilization paradigms, and pharmacological mechanisms.

A recent review by Prakash et al. consolidates its phytochemical and pharmacological profile, emphasizing properties such as antioxidant, anticancer, hepatoprotective, and antimicrobial activities (Prakash et al., 2024). It also provides a comprehensive overview of commercial formulations derived from various plant parts (e.g., berries, seeds, leaves) alongside their respective clinical or industrial applications. Systematic phytochemical and pharmacological studies have identified a complex array of bioactive constituents in *H. rhamnoides*, including tocopherol isomers, flavonoid glycosides, pentacyclic triterpenes, steroidal alkaloids, bioactive peptides, omega-7 fatty acid derivatives, phenolic acids, and oligosaccharides. This phytochemical profile imparts pleiotropic bioactivities, such as regulating inflammatory cascades, reducing oxidative stress, alleviating hepatotoxicity, improving cardiovascular pathologies, delaying senescence, enhancing immune surveillance, suppressing oncogenesis, and inhibiting microbial pathogenesis (Figure 1). A recent review by Żuchowski systematically summarizes advances in the phytochemistry and pharmacology of *H. rhamnoides* from 2010 to 2021, highlighting the identification of dozens of novel phenolic compounds, triterpenoid saponins, and flavonolignans (Żuchowski, 2023). These discoveries further underscore the plant’s considerable chemical diversity and untapped therapeutic potential in nutraceutical and pharmacotherapeutic development.

A comprehensive literature search was undertaken across major global and regional electronic databases (PubMed, Web of Science, CNKI, Google Scholar) from their respective inceptions to April 2025, employing a combination of Medical Subject Headings (MeSH) terms, keywords, and Boolean logic combinations (e.g., “*Hippophae rhamnoides* AND phytochemistry”) for systematic retrieval of relevant studies. Explicit predefined inclusion criteria encompassed original research articles, clinical trials, and systematic reviews/meta-analyses containing original data on the phytochemistry, pharmacology, or clinical applications of *H. rhamnoides*, published in English or Chinese with robust methodological documentation. Exclusion criteria included publications in non-English/Chinese languages, studies with insufficient methodological rigor or high risk of bias, narrative reviews lacking original data or analytical insights, and works focusing solely on agronomic cultivation, ecological conservation, or industrial processing without relevance to phytochemical or pharmacological research. To mitigate potential regional and publication biases, the database selection integrated international and Chinese academic platforms to ensure global research representation; no restrictions were imposed on study origin, sample size, or publication status (encompassing peer-reviewed journals, conference proceedings, and doctoral/master’s theses); and two independent reviewers with expertise in ethnopharmacology and pharmacology conducted a two-step screening process (initial title/abstract assessment, followed by full-text eligibility evaluation). Discrepancies between reviewers were resolved through consensus or consultation with a senior reviewer specializing in botanical medicine. This rigorous, transparent search and selection protocol facilitated the synthesis of high-quality, unbiased evidence, affording a robust foundation for the critical evaluation of *H. rhamnoides*’ ethnomedicinal legacy, contemporary applications, phytochemical profile, and molecular pharmacodynamic mechanisms.

## 2 Ethnopharmacological uses of *Hippophae rhamnoides L.*

*Hippophae rhamnoides* represents a botanical resource of substantial ethnomedicinal importance, with a documented history of use spanning millennia across diverse traditional medical systems. Within Sino-Mongolian ethnomedical traditions (Liang and Zhao, 2019), this species is formally recorded in several canonical pharmacopeias. These include the Tibetan *rGyud-bzhi* (Four Medical Tantras) (Dunzhu, 1996), the Mongolian *Mongγol udq-a üsüg-ün čindamani erike* (White Crystal Beads of Pharmaceutical Identification), and Li Shizhen’s *Bencao Gangmu* (Compendium of Materia Medica) (Wang et al., 2012). Its traditional therapeutic applications primarily address bronchopulmonary ailments, functional gastrointestinal disorders, thermal injuries, and various hematological abnormalities. *Hippophae rhamnoides* also holds a significant position in broader Eurasian ethnopharmacological practices. Documented applications include its zootechnical use in Hellenic animal husbandry, deployment for ecological rehabilitation in German land management, and utilization in dermatological and gastroenterological therapeutics within Russian and Central Asian traditional medicine (Suryakumar and Gupta, 2011). Despite this extensive and enduring legacy, contemporary research on *H. rhamnoides* faces significant knowledge gaps. These include a lack of phytochemical standardization, insufficient elucidation of mechanistic pharmacodynamics, and underdeveloped clinical translation protocols. Furthermore, rigorous scientific validation and cross-cultural comparative analysis of its ethnomedical claims remain notably inadequate.

This review provides a comprehensive overview of the ethnopharmacological applications of *H. rhamnoides* in Sowa-Rigpa (Tibetan), Mongolian, Traditional Chinese, and broader Eurasian medical systems, while offering a critical analysis of its underlying pharmacodynamic mechanisms. The principal findings are threefold: (1) In Sino-Mongolian medical traditions, *H. rhamnoides* is primarily employed to regulate the splenic-gastric axis, suppress cough, and improve hemorheology, with notable consistency observed in its documented materia medica properties (e.g., nature, flavor, channel tropism) across these systems. (2) Eurasian applications predominantly emphasize dermatological therapies, management of gastrointestinal pathologies, and pedospheric rehabilitation, underscoring the plant’s functional adaptability across diverse anthropogenic ecosystems. (3) Contemporary pharmacological studies mechanistically validate its ethnomedical uses and clarify its anti-inflammatory, antioxidant, and tissue-regenerative properties, including the acceleration of epithelial restitution. Collectively, these insights clarify the molecular basis of *H. rhamnoides*’ therapeutic efficacy and establish a foundational framework for its future phytopharmaceutical standardization and translational clinical development.

### 2.1 Ethnopharmacological deployment in Sino-Tibetan medicine

#### 2.1.1 *Sowa-Rigpa* (*Tibetan medicine*) praxis

As a core medicinal material in the Tibetan medicine system, *H. rhamnoides* has its medicinal practice mainly concentrated in the Qinghai-Tibet Plateau and surrounding Tibetan-inhabited areas (Dunzhu, 1996). Its medicinal value is systematically recorded in classic works such as *Yue Wang Yao Zhen (King’s Medicine Diagnosis)*, *Si Bu Yi Dian (Four Tantras of Tibetan Medicine)*, and *Jing Zhu Ben Cao (Crystal Pearl Materia Medica)*. Characterized by a sour and slightly sweet taste, and a warm, dry, and sharp nature, its efficacy is deeply consistent with the Tibetan medical theory of balancing the three humors (Bumei, Chiba, Long), forming a regulatory system for multi-system disorders.

Its effect of benefiting the lung and resolving phlegm is particularly prominent: targeting viscous phlegm in the lungs and throat, it serves as a common medicine for respiratory diseases such as lung abscess, pulmonary tuberculosis, emphysema, and tracheitis, and can effectively relieve symptoms including cough, asthma, and laryngeal obstruction. Its efficacy of promoting blood circulation to remove blood stasis and dredging collaterals is applicable to cardiovascular diseases such as hyperlipidemia, coronary heart disease, and angina pectoris, as well as blood stasis-related conditions like traumatic injuries and hematomas; meanwhile, it also relieves gynecological blood stasis accumulation such as amenorrhea, irregular menstruation, and uterine fibroids. Its ability to invigorate the spleen, harmonize the stomach, and stimulate appetite promotes food digestion and absorption, regulates digestive system diseases including indigestion, superficial gastritis, atrophic gastritis, gastric ulcer, and liver disorders, and improves digestive disturbances such as epigastric dull pain, abdominal distension, and hiccups (Dunzhu, 1996).

The efficacy of enhancing body yang and tonifying blood can moisturize the skin, promote hair growth, and is suitable for the conditioning of frail populations such as the elderly and emaciated individuals, while helping to reduce the incidence of breast and laryngeal diseases. In addition, its effects of reducing swelling, relieving pain, detoxifying, and protecting healthy qi can assist in the treatment of carbuncles, furuncles, diphtheria, edema, tumors, plagues, poisoning, and parasitic diseases. It has no obvious toxic or side effects except for mild impacts on the gallbladder (with the exception of excessive consumption). In clinical application, *H. rhamnoides* is mostly used by collecting fruit juice to make ointments or combining with other medicines into powders (e.g., Wu Wei Sha Ji San [Seabuckthorn Five-Flavor Powder], Sha Ji Gao [Seabuckthorn Ointment]). Its traditional efficacy and treated diseases mutually confirm, constructing a complete medicinal system based on Tibetan medical theory (Dunzhu, 1996).

#### 2.1.2 Mongolian ethnopharmacology praxis

Mongolian medicine has a profound theoretical accumulation and practical inheritance in the application of *H. rhamnoides*, with relevant records in classic works such as *Yin Shan Zheng Yao (Dietary Guidelines for Imperial Physicians)*, *Ren Yao Bai Jing Jian (Crystal Mirror of Materia Medica Identification)*, and *Meng Yao Zheng Dian (Authentic Mongolian Materia Medica)* (Liang and Zhao, 2019). Known as “Chiqirigan” in Mongolian, *H. rhamnoides* is characterized by a sour and astringent taste, and possesses warm, dry, oily, and astringent properties. Its medicinal efficacy is closely linked to core pathogenesis in Mongolian medicine such as “Badagan” (phlegm-dampness syndrome) and “Baoru disease”, showing strong targeting in clinical application.

Relieving cough, resolving phlegm, and clearing lung heat is one of its core efficacies: it mainly treats respiratory diseases such as excessive phlegm, chronic bronchitis, emphysema, and lung abscess, and can alleviate symptoms like dyspnea and difficulty in expectoration. The record of “promoting fluid production, quenching thirst, and relieving cough” in *Yin Shan Zheng Yao* confirms its effect in relieving cough and promoting fluid production. Clinically, it is often combined with raisins, licorice, and other medicines to prepare formulations such as Sha Ji Wu Wei San (Seabuckthorn Five-Flavor Powder) and Ba Yage Qi Wei San (Bayage Seven-Flavor Powder), which are used for lung-heat cough, whooping cough, and other conditions. Its efficacy of promoting blood circulation to remove blood stasis, regulating menstruation, and relieving pain is applicable to gynecological diseases caused by blood stasis obstruction such as blood mass, amenorrhea, retained placenta, and lower abdominal pain, as well as traumatic swelling and visceral blood stasis. *Ren Yao Bai Jing Jian* records its “blood circulation-promoting and lung abscess-resolving” effects; formulations such as Sha Ji Shi Qi Wei San (Seabuckthorn Seventeen-Flavor Powder) and Huo Xue Liu Wei San (Blood-Circulating Six-Flavor Powder) further enhance the efficacy of regulating menstruation and resolving blood stasis by combining with borax, safflower, and other ingredients (Liang and Zhao, 2019).

Moreover, its ability to invigorate the spleen, aid digestion, harmonize the stomach, and relieve pain can improve digestive system problems including indigestion, stomachache, abdominal distension, and food stagnation-induced abdominal pain. *Meng Gu Yi Xue Jing Dian Cong Shu Yao Wu Xue (Mongolian Medical Classic Series Pharmacology)* clearly confirms its “digestion-aiding” effect; single preparations such as Sha Ji Jing (Seabuckthorn Extract) and Sha Ji Gao (Seabuckthorn Ointment) can be directly used for such conditions, and *H. rhamnoides* oil has a certain preventive and therapeutic effect on pylorus-ligated, stress-induced, and other types of gastric ulcers. In addition, its ability to dispel “Badagan” and detoxify protects healthy qi, which can address “Badagan”-related diseases and Baoru disease. The statement in Meng Yao Zheng Dian that “*H. rhamnoides* resolves phlegm, dissolves blood stasis, and treats Badagan disease” highlights its core value; its stem extract and ash also have blood circulation-promoting, heat-clearing, and stinging pain-relieving effects, which can assist in alleviating visceral and intestinal stinging pain. The aforementioned efficacies and applications are all included in modern Mongolian medical monographs such as *Nei Meng Gu Meng Cheng Yao Biao Zhun (Inner Mongolia Mongolian Patent Medicine Standards)* and *Meng Yi Yao Fang Hui Bian (Compilation of Mongolian Medical Prescriptions)*, forming a complete application system based on Mongolian medical theory(Liang and Zhao, 2019).

#### 2.1.3 Pharmacognosy in traditional Chinese medicine

Traditional Chinese medicine’s understanding of *H. rhamnoides*’s medicinal value is concentrated in *Ben Cao Gang Mu (Compendium of Materia Medica)* compiled by the renowned Ming Dynasty pharmacologist Li Shizhen (1518–1593), whose records provide an important theoretical basis for the traditional application of *H. rhamnoides* (Wang et al., 2012). The nature and flavor characteristics of *H. rhamnoides (“sour, warm, and non-toxic”)* determine its mild medicinal properties and relatively wide application scope, with core applications focusing on the regulation of digestive system diseases and the improvement of fluid metabolism.

Relieving diarrhea, killing parasites, regulating qi, and reducing distension are its core efficacies: it is suitable for symptoms such as chronic intractable dysentery, epigastric and abdominal fullness, and emaciation with sallow complexion, and is particularly effective against tapeworm (“cunbai chong”) infection. The traditional usage is to grind it into powder alone and take one qianbi (a traditional Chinese medicinal measurement, approximately 1.5–3 g) mixed with wine. Meanwhile, its efficacy of promoting fluid production, moistening dryness, sobering up, and quenching thirst can improve dry mouth caused by fluid deficiency and thirst discomfort after drinking alcohol; eating it after pickling with salt or vinegar can further enhance the fluid-promoting effect. The mild and non-toxic nature of *H. rhamnoides* provides safety support for its clinical application (Wang et al., 2012). Although the recorded content is relatively concise, it forms a practical medicinal system characterized by “clear efficacy, specific usage, and targeted indications”, reflecting the precise grasp of seabuckthorn’s medicinal value in traditional Chinese medicine.

#### 2.1.4 Comparative analysis across traditional medical systems

To systematically reconcile the differences across Sowa-Rigpa (Tibetan medicine), Mongolian medicine, and Traditional Chinese Medicine while maintaining a coherent comparative logic, this section centers on the “efficacy-disease correspondence” as the unifying framework. Rooted in the three humors theory (Bumei, Chiba, Long), Tibetan medicine characterizes *H. rhamnoides* with a warm, dry, and sharp nature, emphasizing its pleiotropic regulatory effects on multi-system disorders, particularly lung-benefiting and phlegm-resolving actions for respiratory conditions (e.g., lung abscess, pulmonary tuberculosis) and blood circulation-promoting effects for cardiovascular and gynecological stasis-related diseases, which align with its role in balancing humoral imbalance. In contrast, Mongolian medicine, based on the “three roots and seven elements” theory, links *H. rhamnoides* (known as “Chiqirigan”) with addressing core pathogeneses such as “Badagan” (phlegm-dampness syndrome) and “Baoru disease,” with core efficacies focusing on lung-heat clearing and phlegm-resolving for chronic bronchitis, blood stasis-dispersing for gynecological disorders (e.g., amenorrhea, retained placenta), and spleen-invigorating for digestive disturbances, often enhanced by combinations with borax and safflower in formulations. TCM, grounded in the five elements theory and recorded in *Compendium of Materia Medica*, describes *H. rhamnoides* as “sour, warm, and non-toxic,” with a more focused application on digestive system regulation (e.g., relieving intractable dysentery, tapeworm infection) and fluid metabolism improvement (e.g., quenching thirst, sobering up), featuring concise yet targeted usage such as powder taken with wine. Despite these differences in theoretical underpinnings and terminology, all three medical systems converge on *H. rhamnoides*’ efficacy in regulating digestive and respiratory functions, reflecting a shared recognition of its therapeutic value rooted in regional clinical practices.

### 2.2 Ethnobotanical utilization in Eurasian traditions

*Hippophae rhamnoides* demonstrates significant ethnobotanical value across Eurasian ecoregions. In Hellenic animal husbandry (Suryakumar and Gupta, 2011), its leaf and branch biomass is utilized as a feed supplement to support digestive health, showing pronounced efficacy in *Equus caballus* (horse) populations. Concurrently, German land rehabilitation protocols employ this species for soil remediation, specifically for the ecological restoration of post-industrial derelict sites and colliery spoils through its robust root system, which facilitates rhizospheric and geomorphic stabilization. Furthermore, empirical evidence shows that dietary supplementation with its fruit pomace in ovine (*Ovis aries*) models exerts neutral effects on key serum metabolic parameters, including glucose, triglycerides, and HDL/LDL cholesterol levels, as well as on the retention of essential nutrients (e.g., crude protein and lipids) and organoleptic meat quality (Qin et al., 2020). Crucially, such supplementation enhances myofibrillar protein deposition, optimizes textural attributes, improves water-holding capacity, and increases oxidative stability in the meat. These findings collectively position *H. rhamnoides* pomace as a viable phytogenic feed additive for sustainable sheep production systems.

### 2.3 Limitations of ethnopharmacological research on *Hippophae rhamnoides*

Despite the comprehensive summary of ethnopharmacological uses across different medical systems, this section has several inherent limitations that warrant critical reflection. First, the documentation of Tibetan medicine’s application of *H. rhamnoides* relies heavily on classic materia medica (e.g., *Four Tantras of Tibetan Medicine*) with limited contemporary clinical validation data, making it difficult to assess the consistency of traditional efficacy in modern populations (Dunzhu, 1996). Second, Mongolian medicine-related research is predominantly concentrated in Inner Mongolia and surrounding Mongolian-inhabited areas (Liang and Zhao, 2019), lacking sufficient data from other Mongolian-inhabited regions across Eurasia, which may introduce regional bias in characterizing its ethnopharmacological spectrum. Third, TCM’s records of *H. rhamnoides*, while concise and practical, are relatively fragmented in *Compendium of Materia Medica* and other classics, with insufficient systematic exploration of its mechanism of action within the TCM theoretical framework (e.g., the correlation between its “sour-warm” nature and meridian tropism) (Wang et al., 2012). Fourth, the ethnobotanical uses in Eurasian traditions, such as its application as animal feed in Hellenic husbandry and soil remediation in Germany, are primarily based on empirical observations or small-scale practical trials, lacking large-scale controlled studies to validate their efficacy and safety. These limitations highlight the need for future cross-regional, multi-center clinical studies and in-depth mechanistic research to complement and verify the traditional ethnopharmacological evidence of *H. rhamnoides*.

## 3 Modern applications of *Hippophae rhamnoides L.*

*Hippophae rhamnoides* represents a phytogenic resource of considerable promise, with rapidly expanding applications in modern industrial and therapeutic sectors (Prakash et al., 2024). Recent multidisciplinary research has accelerated the translation of its traditional uses into diversified industrial domains, including food processing, dermocosmetics, animal husbandry, and environmental remediation. The plant’s diverse profile of bioactive constituents, particularly flavonoids, essential vitamins, and unsaturated fatty acids, confers multifunctional properties such as mitigating oxidative stress, exerting anti-inflammatory effects, and modulating immune homeostasis. These mechanistically elucidated bioactivities provide a scientifically substantiated foundation for its broad-scale industrial utilization.

### 3.1 Novel applications in animal nutrition systems

Data from Qin et al. shows that dietary supplementation with *Hippophae* pomace has no significant impact on most serum biomarkers, key nutritional parameters, or essential meat quality traits in ovine models (Qin et al., 2020). Concurrently, it enhances muscular development, improves tenderness, increases water-holding capacity, and elevates oxidative stability. These combined effects establish it as a viable phytogenic supplement for sustainable sheep production systems.

### 3.2 Potential nutraceutical applications

The nutritional density of *H. rhamnoides* berries, characterized by high levels of ascorbic acid, tocopherols, and carotenoids, supports their utilization in functional foods and nutraceutical beverages (Gâtlan and Gutt, 2021; Wang et al., 2022a). Commercial food products fortified with *Hippophae* include fruit-blended dairy composites, frozen desserts, baked goods, fermented yogurts, preserved spreads, and gel-based confections. Beverage derivatives consist of pressed juices, fermented wines, malted grain infusions, and aqueous botanical extracts. These formulations exhibit favorable organoleptic properties and deliver pleiotropic physiological benefits, such as enhanced immune function and reduced oxidative stress.

Growing concerns over the toxicological profiles of synthetic antioxidants (Feng et al., 2023) have intensified the development of effective natural alternatives as a priority in food additive innovation. In this context, researchers have investigated the synergistic potential of *H. rhamnoides* leaf flavonoids with chitosan. A notable application is a hybrid freshness-preserving formulation engineered for minimally processed lettuce, which incorporates *Hippophae* foliar flavonoids and chitosan polymers. This formulation demonstrates significant preservation efficacy, showing substantial potential for enhancing microbiological stability and extending product shelf life.

### 3.3 Dermocosmetic formulations

Dermocosmetic formulations incorporating bioactive fractions of *H. rhamnoides* are commercially available in global markets (Gâtlan and Gutt, 2021). The product spectrum includes topical applications such as humectant emulsions, rejuvenating bio-cellulose matrices, trichological serums, and various cleansing agents, ranging from surfactant-based cleansers to topical emulsions and epidermal detergents. This utilization has been further extended to oral care systems, including phytogenic mouth rinses.

### 3.4 Pharmacotherapeutic formulations

*Hippophae rhamnoides* serves as the source for a range of standardized phytopharmaceutical preparations. It maintains enduring prominence in various pharmacopeias, grounded in ethnomedical practice and increasingly validated by evidence-based pharmacological research. This is reflected in the wide array of commercial formulations available in markets across China and internationally (Prakash et al., 2024), a development that underscores the successful industrial translation of this botanical resource. Key formulations, systematically categorized below with reference to contemporary pharmacopeial standards (Wang et al., 2012), illustrate this translation.

#### 3.4.1 Cardiometabolic therapeutics

Pharmaceutical preparations targeting cardiovascular pathologies include *Hippophae*-based formulations such as Xindakang tablets and encapsulated supplements. These therapeutics are known to mediate hypolipidemic effects, hemodynamic modulation, and microcirculatory enhancement, thereby mitigating pathological progression in cardiovascular disorders (Wang et al., 2012).

#### 3.4.2 Respiratory afflictions

Therapeutic interventions for respiratory conditions employ *Hippophae*-derived pharmacopeial preparations, including granular composites, pulverized Wuwei Shaji formulations, buccal troches, and compressed tablets. These formulations provide symptomatic relief through antitussive actions, sputum rheology modulation, and bronchial smooth muscle relaxation, thereby supporting respiratory tract homeostasis (Wang et al., 2012).

#### 3.4.3 Gastrointestinal disorders

For gastrointestinal disorders, Hippophae-based therapeutics with established applications include lyophilized emulsion preparations, anti-erosive powder formulations, oleaginous oral suspensions, and enteric-coated soft gelatin capsules. These agents mediate gastrointestinal motility modulation, enhance nutrient assimilation, and ameliorate gastralgia and gastric acid hypersecretion (Wang et al., 2012).

#### 3.4.4 Reproductive system pathologies

For the treatment of reproductive system pathologies, *Hippophae*-integrated therapeutics, including Ershiwuwei Guijiu polyherbal tablets, Shiyiwei Nengxiao encapsulated formulations, and Shenqi Shaji composite solutions, offer targeted therapeutic regimens. These preparations exhibit properties that potentiate gonadal function and regulate the menstrual cycle, thereby supporting urogenital homeostasis (Wang et al., 2012).

#### 3.4.5 Dermatological disorders

For dermatological disorders, topical formulations, such as Shuanghuang *Hippophae*-Eucalyptus emollient ointment, demonstrate established clinical utility. These agents deliver anti-inflammatory, anti-infective, anti-pruritic, and analgesic effects, while facilitating tissue regeneration and improving integumentary status (Wang et al., 2012).

Collectively, the pharmacopeial spectrum of *Hippophae*-derived agents encompasses considerable diversity, spanning therapeutic applications across cardiovascular, respiratory, gastrointestinal, and dermatological pathologies. As scientific inquiry progresses and technological innovations emerge, the therapeutic repertoire and clinical utility of *Hippophae*-based formulations are projected to expand, thereby enhancing their impact on global health initiatives.

### 3.5 Ecophysiological remediation

*Hippophae rhamnoides* functions as a keystone species in the rehabilitation of degraded ecosystems (Liu et al., 2024). It exhibits exceptional xerophytic adaptations and oligotrophic tolerance, alongside cryoprotective and anhydrobiotic mechanisms that enable it to thrive in arid environments. Its actinorhizal symbiosis with *Frankia* spp. facilitates substantial atmospheric nitrogen fixation, thereby accelerating pedogenesis through organic matter accumulation and soil structure regeneration. Following its establishment, shrub and grass diversity increases markedly, promoting the development of stable plant communities that enhance faunal biodiversity and provide critical support for endangered species conservation. Furthermore, the plant’s extensive root system effectively reduces Hortonian overland flow and sediment transport, increases soil water-holding capacity, and mitigates aeolian erosion, serving as an efficient biogeomorphic barrier against desertification. Notably, *H. rhamnoides* has been widely applied in soil erosion control and desertification prevention in China (such as the 1.35 million mu plantation in Ordos that reduces 4.5 million tons of sediment into the Yellow River annually (Gao et al., 2024)), with over 8 million mu of artificial seabuckthorn forests in the Shanxi-Shaanxi-Inner Mongolia soft sandstone area (Pisha Yan area) forming the world’s largest ecological restoration project(Li et al., 2023).

### 3.6 Radioprotective applications in space environments

*Hippophae rhamnoides* demonstrates considerable potential for mitigating the effects of cosmic radiation, a property attributed to its unique phytoconstituents which exhibit synergistic radioprotective and antioxidant activities (Gâtlan and Gutt, 2021). These bioactive compounds are postulated to mitigate radiation-induced cellular damage through mechanisms involving free radical scavenging and the modulation of DNA repair pathways. Further empirical validation under simulated spaceflight conditions is warranted to substantiate its applicability for radioprotection in aerospace contexts.

To clarify the clinical and commercial validation status of *H. rhamnoides*’s modern applications, it is noteworthy that nutraceuticals, dermocosmetics, pharmacotherapeutic formulations, and ecophysiological remediation have reached mature stages: nutraceuticals are globally marketed as juices, dairy products, and supplements (with the chitosan-flavonoid preservative still in laboratory stages); dermocosmetics have been commercially validated for decades across skincare and oral care categories; pharmacotherapeutics are listed in pharmacopeias and commercially available with proven clinical efficacy; and ecological remediation has been confirmed through large-scale engineering projects. In contrast, animal nutrition applications, while effective in ovine models, lack large-scale industrial adoption due to the need for dosage and cost optimization, and space radioprotective applications remain experimental without simulated spaceflight validation.

Beyond the validation disparities of its diverse applications, additional critical considerations constrain the large-scale translation of *H. rhamnoides* into nutraceuticals and pharmaceuticals: contaminant surveillance and regulatory classification ambiguity. First, contaminant surveillance is imperative. As with all botanical materials, *H. rhamnoides* products are susceptible to contamination with heavy metals, pesticide residues, mycotoxins, and microbial pathogens. Given the plant’s widespread cultivation for ecological restoration in degraded areas (Section 3.5), which may include sites with industrial legacy contamination, and its documented exceptional stress tolerance and oligotrophic adaptations, the risk of heavy metal bioaccumulation in edible tissues warrants rigorous monitoring. The lack of internationally harmonized contaminant limits specific to *H. rhamnoides* products represents a regulatory gap; while Current Good Manufacturing Practice (cGMP) compliance and systematic testing protocols are essential, they are not universally implemented across global supply chains. Second, regulatory classification ambiguity poses commercial and translational challenges. As explicitly discussed in the Introduction, the term “nutraceutical” lacks legal recognition as a distinct category under international regulatory frameworks (Santini et al., 2018; Thakur et al., 2024). The U.S. Food and Drug Administration (FDA) and European Food Safety Authority (EFSA) classify such products as “food supplements” or “functional foods” rather than “nutraceuticals,” and despite efforts to standardize the terminology, no globally uniform definition has been adopted. This regulatory patchwork, with products categorized as dietary supplements (U.S.), food supplements (E.U.), or traditional herbal medicines (various Asian countries), complicates international commercialization, imposes inconsistent label claim restrictions, and fragments post-market surveillance requirements. For *H. rhamnoides*, this ambiguity particularly hinders the translation of traditional ethnopharmacological uses (extensively documented in Sections 2.1-2.2) into legally permissible health claims. Collectively, these validation disparities, contaminant risks, and regulatory ambiguities underscore the need for targeted research, harmonized international standards, and standardized quality control systems to fully unlock the industrial and clinical potential of *H. rhamnoides*.

## 4 Phytochemical architecture of *Hippophae rhamnoides L*

A recent comprehensive review by Żuchowski corroborates the considerable phytochemical diversity of *H. rhamnoides*, reporting the identification of more than 24 novel flavonoids, 10 flavonolignans, 6 saponins, and several alkaloids and terpenoids between 2010 and 2021 (Żuchowski, 2023). Scientific interest in *Hippophae* species has grown substantially in nutraceutical and pharmacotherapeutic research, with fruits (drupes), seeds, and leaves serving as the primary reservoirs of bioactive compounds. These organs exhibit distinct phytochemical profiles that warrant further biotechnological exploration. This review systematically outlines the spectrum of phytoconstituents characteristic of each organ, elucidates their structure-activity relationships and biofunctional properties, and highlights current research directions. By integrating this knowledge, the review aims to facilitate the unlocking of latent therapeutic and industrial potential, thereby establishing a mechanistic foundation for the targeted utilization of *H. rhamnoides* across interdisciplinary applications.

### 4.1 Drupaceous bioactive phytochemicals

The drupe serves as the principal phytochemical reservoir in *Hippophae* species, exhibiting high nutraceutical density and marked biofunctional properties that garner considerable interest in functional food development and pharmacognosy research. According to Dubey et al., 100 g of dried *H. rhamnoides* berries provide approximately 275 kcal, containing 55.4 g carbohydrates, 14.1 g fiber, 7.1 g fat, and 3.7 g protein, in addition to substantial quantities of vitamin C, vitamin E, beta-carotene, and essential minerals, thereby corroborating its nutritional and functional value (Dubey et al., 2024). Phytochemical analyses identify a diverse range of bioactive constituents, including phenolic compounds, saccharides, essential vitamins, volatile terpenoids, structural lipids, carotenoid pigments, organic acids, proteinogenic amino acids, and essential minerals. Notably, significant cultivar-dependent variations in these phytochemicals have been documented (Criste et al., 2020). For instance, Criste et al. demonstrated that among four Romanian sea buckthorn cultivars, the SF6 genotype yielded the highest total phenolic and flavonoid content in berries, whereas leaf extracts consistently contained higher polyphenol levels than berries, underscoring the substantial influence of genetic background on phytochemical composition. This phytocomplex not only supports the fruit’s organoleptic properties and dietary applicability but also shows substantial pharmacotherapeutic potential, laying a foundation for evidence-based drug discovery. A comprehensive structural elucidation and phytochemical characterization is systematically presented in Supplementary Table 1.

*Hippophae rhamnoides* exemplifies a quintessential dual-purpose botanical resource, with its drupes exhibiting a multifaceted pharmacodynamic profile. Empirical studies have revealed a range of pleiotropic bioactivities, such as mitigating oxidative stress, delaying senescence, modulating immune homeostasis, exerting anti-carcinogenic potential, enhancing the cutaneous barrier, suppressing microbial pathogenesis, and maintaining gastrointestinal equilibrium. These properties substantiate its therapeutic relevance in preventive healthcare and wellness preservation strategies. The molecular mechanisms underlying these bioactivities have been progressively elucidated through rigorous mechanistic studies, with the interrelationships of key signaling pathways systematically summarized in Table 1. This review methodically examines the phytochemical and pharmacological nexus of *H. rhamnoides* drupes, integrating current research to establish a conceptual framework for targeted bioprospecting and evidence-based applications.

**TABLE 1:** Pharmacological effects of *Hippophae rhamnoides* berry bioactives.

Active phytochemicals	Model	Dose	Pharmacological effects	Mechanism of action	References citations
Phenolic phytochemicals	*In vitro*	50 μg/mL	Suppression of hydrogen peroxide or Fenton reaction-mediated plasma lipid peroxidation and protein carbonylation.	Mediated through free radical neutralization, transition metal sequestration, and lipid peroxidation cascade suppression;	Olas et al. (2016), Mohamed et al. (2020)
Carotenoids	*In vitro*	0.25–75.90 mg L^−1^	2,2-Diphenyl-1-picrylhydrazyl radical quenching capacity	Synergistic antioxidant potentiation with tocopherol isomers;	Górnaś et al. (2016)
Polysaccharides	*In vivo*	200 mg/kg/body	Attenuation of malondialdehyde accumulation in murine models; Enhancement of superoxide dismutase biosynthesis.	Through modulation of redox homeostasis biomarkers	Nowak et al. (2018)
Carotenoid pigments	*In vitro*	35 mg/mL enzyme	Inhibition of acetylcholinesterase enzymatic activity; Profound inhibition of butyrylcholinesterase enzymatic activity	Putative suppression of neurodegeneration-associated enzymatic targets	Tkacz et al. (2020)
Flavonoids	*In vivo*	​	Enhanced leukocyte proliferation and humoral immune responsiveness in senescent models; Upregulation of immunoglobulin isotypes and interleukin-mediated immune signaling	Mononuclear phagocyte system activation	Dannenberger et al. (2018)
Structural polysaccharides	*In vivo*	400 μg/mL	Potentiation of macrophage phagocytic capacity	Immunocyte functional potentiation	Wang et al. (2020)
n-3 Polyunsaturated fatty acids	*In vitro*	​	Modulation of pro-inflammatory gene expression with chronic inflammation resolution	Pomace-mediated inflammatory response modulation	Guo et al. (2017b)
Isorhamnetin	*In vitro*	2 μM	Suppression of mast cell degranulation and eicosanoid mediator release; Inhibition of calcium ion transmembrane flux	Type I hypersensitivity mitigation	Qiu et al. (2023)
Total flavonoid content	*In vitro*	10 μg/mL	Downregulation of pro-inflammatory cytokine cascades; NF-κB signal transduction pathway blockade	p38/JNK/NF-κB signalosome-mediated inflammation resolution	Li et al. (2016), Jiang et al. (2017), Ren et al. (2019)
Bioactive polysaccharides	*In vivo*	200, 100, 50 mg/kg/day	Hypoglycemic and anti-inflammatory efficacy in diabetic models	Inflammatory cell infiltration dynamics regulation	Yuan et al. (2016)
Bioaccessible polyphenols	*In vitro*	IC_50_ ≤ 1.66–9.22 mg/mL	Cytostatic potency against neoplastic cell lineages	Flavanol-mediated neoplastic proliferation suppression	Guo et al. (2017a)
Total flavonoid profile	*In vitro*	35 μM	CD8^+^ T-lymphocyte mediated tumor microenvironment immunomodulation	Chemokine signaling axis modulation	Hao et al. (2022)
Linoleic acid (fruit oil matrix)	*In vitro*; *In vivo*	10 μL/mL; 2.5 µg/ear	Stratum corneum barrier fortification with transepidermal water loss reduction; Dermatometabolic homeostasis modulation	Oleoresin-induced NF-κB and pro-inflammatory mediator suppression	Balkrishna et al. (2019), Boca et al. (2019)
Palmitic acid (seed oil fraction)	*In vitro*	50 μM	Keratinocyte and dermal fibroblast proliferative stimulation	​	Dudau et al. (2021)
Polyphenol-carotenoid complexes	*In vitro*	500 ng/mL	Ultraviolet radiation-induced cutaneous oxidative damage mitigation	Fibroblast and keratinocyte cytoprotection	Gęgotek et al. (2018)
Polyphenols, polysaccharides, and flavonoids	*In vitro*	A 50 mL of juice was diluted with200 ml HCl	Antimicrobial activity against enteric and cutaneous pathogens; Gut microbiota ecological equilibrium modulation	Beneficial microbiota proliferative stimulation (e.g., Bifidobacterium spp.)	Attri and Goel (2018), Mahajan et al. (2018), Wang and Cao (2023)

### 4.2 Seminal bioactive phytochemicals

The seminal phytoconstituent profile encompasses (Żuchowski, 2023) structural lipids, flavonoid derivatives, phenolic acids, and carboxylic acid derivatives. The structural characterization and compositional distribution of these constituents are systematically presented in Supplementary Table 2. Evidence indicates pluripotent pharmacodynamic properties, including the amelioration of cutaneous xerosis, mediation of adipostatic regulation, provision of hepatoprotective efficacy, enabling of cellular cytoprotection and restitution, manifestation of antioxidant capacities, facilitation of glycemic and lipidemic homeostasis, exertion of retinoprotective efficacy, and mitigation of physical fatigue states. The underlying mechanistic pathways are detailed in Table 2. Nevertheless, the conclusive elucidation of molecular interactions and phytosynergistic mechanisms warrants continued investigation.

**TABLE 2:** Pharmacological effects of *Hippophae rhamnoides* seeds bioactives.

Active phytochemicals	Modle	Dose	Pharmacological effects	Mechanism of action	References citations
Polyunsaturated fatty acid (PUFA)	*In vitro*	10 μg/mL	Reduce skin dryness	Improve skin hydration through increasing AQP3 and HAS2 expressions	Yao et al. (2021)
Flavonoid extracts	*In vivo*	100 mg/kg; 300 mg/kg	Inhibitory effect on obesity to protect the liver	In liver and white adipose tissues, thus helping to reduce fat accumulation	Hao et al. (2020)
Flavonoid extracts	*In vitro*	20 μg/mL	Protects against Alcohol-Induced Intestinal Barrier Dysfunction	Activating the Nrf2-mediated pathway alleviating oxidative stress and enhancing TJ expression	Wei et al. (2023)
Flavonoid, procyanidin, and total phenolic	*In vitro*; *In vivo*	0.1 mg/mL; 100 mg/kg/day.	Cellular protective and reparative effects, antioxidant performance	By reduced apoptosis rates and enhanced resistance to oxidative stress alongside moderate cell repair properties	Hua et al. (2023)
Flavonoids	*In vitro*	10 µM	Relieving Dox-induced cardiac injury and inhibit cardiomyocyte apoptosis.	The mechanisms were mainly related to the stability of mitochondrial structure and function maintained by suppressing the production of intracellular reactive oxygen species (ROS), p-JNK and cleaved caspase-3, and increasing ATP contents and protein expression of mitochondrial mitofusin (Mfn1, Mfn2), Sab and p-Src.	Zhou et al. (2023)
Aqueous extract	*In vivo*	400 mg/kg/day	Hypoglycemic and hypolipidemic effects in the streptozotocin (STZ) and high-fat diet (HFD)-induced type 2 diabetic rats.	Seemed to associate with enhancing insulin sensitivity by decreasing the elevated serum TC and LDL-cholesterol levels in experimental type 2-like diabetic rats	Zhang et al. (2010)
phenolic phytochemicals	*in vitro*	0.5–50 μg/mL; 100 μg/mL	Anti-platelet activity that is not decreased by thermal processing	May be linked to the inhibition of arachidonic acid metabolism and decreasing the exposition of receptors on the surface of platelets	Sławińska et al. (2024)
phenolic phytochemicals	*in vivo*	100 mg/kg/day	Possess a protective effect against light-induced retinal degeneration	Through antioxidant, anti-inflammatory and antiapoptotic mechanisms	Wang et al. (2016)
Fatty acids, vitamin E, and phytosterols	*In vivo*	23.6%(w/w)	Has potential to ameliorate non-alcoholic fatty liver diseases (NAFLD), and related metabolic disorders, like obesity and dyslipidemia	By modulating gut microbiota	Wang et al. (2022b)
Phytosterol fatty acids	*In vivo*	4.75% *H. rhamnoides* Seed Oil; 9.5% *H. rhamnoides* Seed Oil	Effective in reducing blood cholesterol in hypercholesterolemic hamsters	*via* increasing intestinal cholesterol excretion and promoting the growth of short chain fatty acids (SCFAs) SCFAs-producing bacteria.	Hao et al. (2019)
*H. rhamnoides* seed oil	*In vivo*	0.85, 1.68, 3.35 g/kg	Antifatigue effect	May be related to its inhibition of oxidative and inflammatory injury and regulation of hypothalamic neurotransmitters	An et al. (2023)
*H. rhamnoides* seed polyphenols	*In vitro*	0.25–0.5 mg/mL	Preservation effect natural, biological preservative.	The scavenging activitys of free phenol on DPPH, ABTS and hydroxyl free radicals, superoxide anions and nitrite were close to VC, and it had strong inhibitory ability against *Staphylococcus aureus* (*S. aureus*).	Huang et al. (2023)

### 4.3 Foliar bioactive phytochemicals

*Hippophae* foliage demonstrates multifaceted applications across pharmacotherapeutic, nutraceutical, and zootechnical domains, harboring abundant bioactive phytochemicals, including (Żuchowski, 2023) flavonoid glycosides, structural polysaccharides, proteinogenic amino acids, essential vitamins, polypeptide complexes, and dietary fiber fractions. Analytical evidence confirms superior flavonoid and polyphenolic density relative to drupaceous organs, as comprehensively characterized in Supplementary Table 3. It exhibits pluripotent pharmacodynamic properties, including hemodynamic regulation, cholesterostasis, anti-inflammatory and redox-modulatory activities, immunopotentiation, antineoplastic potential, and the management of respiratory conditions. The corresponding mechanistic pathways are detailed in Table 3.

**TABLE 3:** Pharmacological effects of *Hippophae rhamnoides* foliage bioactives.

Active phytochemicals	Modle	Dose	Pharmacological effects	Mechanism of action	References citations
Extract of *H. rhamnoides* leaves	*in vitro*	10%	Treatment of hyperlipidemia	Activate the PPAR signaling pathway, the hypoxia-inducible factor (HIF)-1 signaling pathway, and the AMP-activated protein kinase (AMPK) signaling pathway	Tang et al. (2022)
Extract of *H. rhamnoides* leaves	*In Vitro*	​	Antibacterial and antioxidant	Preventing the formation of microbial biofilms	Tritean et al. (2023)
polysaccharide	*In Vitro*	​	anti-inflammatory	Activate the phagocytic function of macrophages and regulate the TLR4/MyD88/NF-κB signaling pathway	Tanwar et al. (2018)
isorhamnetin	​	​	It has anti-inflammatory effects in chronic obstructive pulmonary disease.	Correct the Th1/Th2 ratio by regulating NF-κB and inhibiting its pathway activity, thereby reducing the expression of inflammatory mediators and asthma-related inflammatory factors.	Chang et al. (2020), Xu et al. (2023)
quercetin	*in vivo*	21.6 mL/kg	Intervene in pulmonary fibrosis rats and cure airway inflammation	Correct the imbalance of Th1/Th2 ratio and improve the function of T lymphocytes	Xing et al. (2020)
Flavonoid extract	*in vitro*	40 µM	anti-inflammatory	Reduced the release of PGE2, NO, IL-6 and IL-8 induced by LPS, activated the Nrf2 signaling pathway and inhibited the LPS-induced inflammation in HGF.	Qi et al. (2018)
Flavonoid extract	*in vivo*	400 mg/kg	Prevention and treatment of PCOS (Polycystic Ovary Syndrome)	Inhibit the inflammatory response mediated by the TLR4/NF-κB signaling pathway	Wang et al. (2024)
ursolic acid	*in vivo*	300 mg/kg/day	Improve the general condition of H_22_ tumor-bearing mice and inhibit the growth of the transplanted tumors	Increasing the concentration of anti-tumor active cytokines such as IL-12 in the blood induces cellular immunity, enhances the body’s immunity, inhibits the degradation of extracellular matrix of liver cancer cells, and suppresses the formation of new blood vessels.	Zhang et al. (2019)
Chokeberry leaf extract	*in vivo*	5%	The healing effect on diabetic wounds in rats	Accelerated the migration of epithelial cells, increasing the levels of hydroxyproline (collagen) and hexosamines	Upadhyay et al. (2025)
Chokeberry leaf extract	*in vitro*	20 mg/mL	Protecting cells	Resist short-wave ultraviolet radiation (The radiation damage caused to HaCaT cells, inhibits cell apoptosis and maintains mitochondrial membrane potential)	Ma et al. (2017)
Chokeberry leaf extract	*In vitro*	100 μg/mL	Inhibition of proliferation of human acute myeloid leukemia cells (KG-1a, HL60 and U937)	Activate the S-phase checkpoint, causing the cell cycle to slow down and promoting cell apoptosis.	Zhamanbaeva et al. (2014)

While the phytochemical richness and pharmacological potential of *H. rhamnoides* (across berries, seeds, and foliage) lay a solid foundation for its development as nutraceuticals or pharmaceuticals, two interrelated challenges significantly impede its translational progress. First, phytochemical standardization remains a significant hurdle. The bioactive composition of *H. rhamnoides* exhibits substantial variability across cultivars, geographical origins, and processing methods. For instance, Criste et al. demonstrated that among four Romanian sea buckthorn cultivars, the SF6 genotype yielded the highest total phenolic and flavonoid content in berries, whereas leaf extracts consistently contained higher polyphenol levels than berries, underscoring the substantial influence of genetic background on phytochemical composition (Criste et al., 2020). Similarly, variations in fatty acid profiles (Dulf, 2012; Vaitkeviciene et al., 2019) and carotenoid contents (Teleszko et al., 2015) have been documented across different cultivars and geographical sources. This heterogeneity complicates the establishment of quality control markers and standardized dosing regimens required by regulatory agencies. Unlike single-molecule pharmaceuticals, botanical extracts containing complex phytochemical mixtures necessitate rigorous analytical protocols to ensure batch-to-batch consistency, a requirement explicitly noted in the need for “phytochemical standardization” identified in this review’s critical analysis of current research gaps.

Second, comprehensive toxicological profiling remains incomplete. While several studies cited in this review report favorable safety profiles, including Upadhyay et al. demonstrating no adverse effects in acute and sub-acute toxicity studies at doses up to 10 mL/kg for 28 days in rats (Upadhyay et al., 2009), and Goel et al. establishing safe radioprotective dosing at 30 mg/kg (Goel et al., 2002), systematic long-term safety data in humans are lacking. The review identifies this as a persistent challenge, noting that “rigorous scientific validation” of ethnomedicinal claims remains “notably inadequate”. Current evidence derives predominantly from traditional use records and animal models, with limited Phase I/II clinical safety trials. The absence of standardized maximum tolerable doses for different extract types (berry, seed, leaf) and administration routes (oral, topical) hinders evidence-based clinical recommendations. These challenges collectively highlight the necessity of targeted research to address phytochemical consistency and long-term safety, thereby bridging the gap between the established pharmacological potential of *H. rhamnoides* and its reliable clinical application.

### 4.4 Critical consideration: Pan-Assay Interference Compounds (PAINS) potential

The rich repertoire of phenolic phytochemicals, particularly flavonoids (e.g., quercetin, isorhamnetin, kaempferol) and tannins, underpins the purported bioactivities of *H. rhamnoides*. However, it is imperative to acknowledge that many of these phytochemicals are classified as PAINS (Magalhães et al., 2021). PAINS are promiscuous molecules that can produce false-positive results in high-throughput *in vitro* assays through non-specific mechanisms rather than targeted biological interactions. Common interference mechanisms include, but are not limited to, aggregation, redox cycling, metal chelation, and chemical reactivity with assay components or protein nucleophiles.

The presence of catechol, quinone, or extensive conjugated systems in many *Hippophae* flavonoids (Supplementary Tables 1, 2, and 3) renders them particularly susceptible to such artifactual activities. Therefore, pharmacological findings derived solely from *in vitro* models utilizing isolated phytochemicals must be interpreted with considerable caution. Claims of specific target engagement based on such assays alone are highly speculative. The biological relevance of these *in vitro* results must be rigorously validated through counter-screening assays, dose-response experiments demonstrating specificity, and, most importantly, *in vivo* studies and clinical trials to confirm genuine therapeutic potential and elucidate specific mechanisms of action. Consequently, subsequent discussions of pharmacological mechanisms must be evaluated through this critical lens.

## 5 Mechanistic pharmacology and critical perspectives on PAINS in *Hippophae rhamnoides L*

*Hippophae rhamnoides*, an archetypal dual-purpose botanical resource, demonstrates multifaceted bioactivity profiles that command escalating scientific scrutiny. Contemporary pharmacodynamic investigations reveal marked therapeutic potential across various physiological domains (Prakash et al., 2024). However, a critical appraisal of this evidence is imperative, particularly for distinguishing robust, specific mechanisms from potential artifacts associated with PAINS prevalent in its phytochemical profile (Magalhães et al., 2021). The elucidation of molecular pathways has advanced through cutting-edge platforms; however, the translational relevance of predominantly *in vitro* findings requires careful evaluation alongside *in vivo* and clinical data to establish a mechanistic foundation for evidence-based clinical implementation.

### 5.1 Hepatoprotective mechanisms

*Hippophae rhamnoides* represents a phytochemical repository with multifunctional bioactivity, and its botanical extracts demonstrate pronounced efficacy in hepatic cytoprotection and fibrotic amelioration. Zhang et al. investigated (Zhang G. et al., 2018) the antifibrotic effects of active phytochemicals from *H. rhamnoides* berries on hepatic stellate cell (HSC) activation and liver fibrosis *in vitro* and *in vivo*. The authors isolated 46 phytochemicals from *H. rhamnoides* and identified three, namely, C13, C15, and C32, with significant inhibitory effects on TGF-β-induced HSC activation. *In vitro*, these phytochemicals reduced inflammatory cytokine levels (TNF-α, IL-1, IL-6), induced cell cycle arrest, and upregulated DNA damage signaling pathways (ATM/ATR-Chk1/Chk2-p53). *In vivo*, the active phytochemicals of *H. rhamnoides* berry (ACSB) were administered at 20 and 40 mg/kg for 4 weeks in a bile duct ligation (BDL) rat model, resulting in attenuated fibrosis, improved liver function, and reduced inflammation.

Guo et al. developed (Guo et al., 2021) a mechanochemical-assisted extraction (MCAE) method to efficiently extract flavonoids from *H. rhamnoides* pomace, achieving a yield of 26.82 mg/g, which is three times higher than that of traditional heat reflux extraction (HRE). The hepatoprotective effects were evaluated using a tetracycline-induced NAFLD model in ICR mice. Animals received intraperitoneal tetracycline (150 mg/kg) for 5 days followed by oral administration of the MCAE-derived extract (MPG) at 200 mg/kg for 15 days, with appropriate control groups: normal control (NCG), disease model (NMG), HRE extract group (HPG), and curcumin control group (CCG) at 200 mg/kg. MPG treatment significantly ameliorated NAFLD-associated biomarkers, including reductions in serum and liver levels of TC, TG, LDL-C, AST, and ALT, an increase in HDL-C, and improved histopathological features such as well-arranged hepatic cords and reduced cytoplasmic vacuolation. The MCAE extract demonstrated superior hepatoprotective effects compared to both the HRE-derived extract and curcumin.

The study by Sheng et al. (2022), investigated the hepatoprotective effects of *H. rhamnoides* sterol (SBS) against CCl_4_-induced acute liver injury in rats, utilizing a combination of biochemical, metabolomic, and transcriptomic approaches. The authors administered SBS at three doses (100, 200, and 400 mg/kg) orally for 7 days prior to CCl_4_ challenge, with bifendate (200 mg/kg) serving as a positive control. The study demonstrated that SBS significantly ameliorated oxidative stress (e.g., increased SOD, GSH-Px, CAT, T-AOC; decreased MDA), reduced inflammatory markers (COX-2, PGE2, γ-GT), and restored metabolic and transcriptional dysregulation.

Based on the critical assessment of the hepatoprotective studies on *H. rhamnoides* extracts, several common limitations and future research priorities emerge, along with important PAINS risks that require emphasis. All three studies lacked comprehensive phytochemical characterization beyond major phytochemicals, with insufficient details on the full spectrum of phytochemicals and their potential synergistic effects. The *in vivo* models used, while relevant, were acute injury models that may not fully recapitulate the complexity of chronic human liver diseases, and all studies omitted crucial *in vitro* mechanistic investigations to elucidate molecular pathways. Furthermore, none established proper dose-response relationships or determined minimum effective concentrations. Future research should prioritize clinical trials to validate efficacy in humans, employ more physiologically relevant chronic disease models, conduct detailed mechanistic studies on key pathways such as Nrf2, NF-κB, and CYP450 regulation, and perform thorough ADMET profiling. Importantly, given the high polyphenol content in *H. rhamnoides* extracts, researchers must be vigilant about PAINS risks, particularly redox activity and assay interference properties that could lead to false positive results in antioxidant and biological assays. These extracts may exhibit promiscuous bioactivity through non-specific mechanisms such as metal chelation, membrane disruption, and protein aggregation, necessitating implementation of rigorous counter-screening assays and hit validation protocols to distinguish true pharmacological activity from assay artifacts. Collectively, *Hippophae* extracts represent promising botanical-derived therapeutics for hepatic pathologies, yet their mechanistic underpinnings warrant further deconvolution. The hepatoprotective effects documented here align with the broader pharmacological profile summarized (Prakash et al., 2024) by Prakash et al. , reinforcing the scientific basis for its traditional use in treating liver disorders.

### 5.2 Antineoplastic

Cancer represents a predominant etiology of global mortality, exhibiting progressively escalating incidence and fatality rates. Carcinogenesis entails multifaceted mechanisms encompassing somatic mutations, dysregulation of signal transduction cascades, and immune evasion strategies. Conventional therapeutic modalities (surgical resection, ionizing radiation, cytotoxic agents, and molecularly targeted interventions) are frequently compromised by adverse events and acquired drug resistance. Consequently, developing innovative antineoplastic agents and therapeutic approaches constitutes a critical biomedical imperative. *Hippophae rhamnoides* demonstrates pharmacologically validated bioactivities spanning antineoplastic efficacy, tumor suppression, and immune homeostasis modulation. The study by Zhang et al. (2019) investigates the antitumor mechanism of polysaccharides extracted from Leucocalocybe mongolica (LMP) in H22 tumor-bearing mice. The research provides a detailed account of the extraction optimization using Response Surface Methodology (RSM), identifying optimal conditions (93 °C, 5 h, 30 mL/g) yielding a polysaccharide content of 6.64%. The polysaccharide composition was characterized *via* LC-MS/MS as a neutral polysaccharide consisting of D-fructose, D-mannose, dextrose anhydrate, D-xylose, trehalose, and galactose. The *in vivo* antitumor activity was assessed using H22 hepatoma-bearing ICR mice, with doses of LMP set at 100, 200, and 300 mg/kg (oral gavage, once daily for 14 days). Cyclophosphamide (CTX, 25 mg/kg) served as the positive control, while saline was used for the negative (model) and normal groups. The study demonstrated significant tumor inhibition rates (75.12%–84.04%) in a dose-dependent manner, alongside immunomodulatory effects (elevated IL-2, IL-6, IFN-γ, TNF-α; reduced VEGF), induction of apoptosis (*via* TUNEL, Bax/Bcl-2, Caspase3), and inhibition of angiogenesis. Liver and kidney function markers (AST, BUN) indicated low toxicity of LMP.

While this study provides valuable *in vivo* evidence for the antitumor activity of Leucocalocybe mongolica polysaccharide (LMP), several limitations should be noted which are common to early-stage pharmacological natural product research. The research lacks *in vitro* assays to elucidate the direct cellular mechanisms of action, leaving the possibility that the observed effects are indirect or mediated by systemic immune modulation. The polysaccharide extract, while optimized for yield, is a crude mixture; its activity could be influenced by minor contaminants or synergistic components, a typical Pitfall of natural product extracts often referred to as PAINS risks in high-throughput screening, though less prevalent in whole-animal studies. The dose range, though effective, was not broad enough to establish a clear minimum effective dose or a full dose-response relationship. Future research should prioritize the purification and structural elucidation of the active polysaccharide fraction(s), employ *in vitro* models to confirm direct cellular targets and exclude assay interference, and investigate the specific immunomodulatory pathways (e.g., TLR/NF-κB) involved. Furthermore, detailed toxicological profiling and pharmacokinetic studies are essential next steps to truly assess its therapeutic potential and overcome the inherent challenges of natural product development.

### 5.3 Neurological disorders

Neurological disorders constitute a spectrum of pathologies involving central and peripheral neural circuitry dysfunction, exemplified by Alzheimer’s disease, Parkinsonism, seizure disorders, and multiple sclerosis. Pathogenesis correlates with neurodegeneration, apoptotic neuronal loss, or synaptic transmission deficits, manifesting as cognitive decline, motor impairment, and sensory processing abnormalities. Current management strategies focus on symptomatic pharmacotherapy and neurorehabilitation but are largely palliative, with inherent limitations such as disease progression and treatment-related adverse events. Consequently, developing disease-modifying interventions represents an urgent neuropharmacological priority. *Hippophae rhamnoides* contains neuroactive phytochemicals with demonstrable therapeutic potential for neurological conditions.

The study by Ladol and Sharma (2021) investigates the neuroprotective effects of *H. rhamnoides* berry extract (Sbt) in an iron-induced epileptic rat model. The extract was administered orally at 1 mL/kg/day for 1 month. The model involved intracortical injection of FeCl_3_ to induce epilepsy, with positive behavioral and electrophysiological outcomes observed. Sbt significantly reduced epileptiform activity, improved memory and anxiety-like behaviors, and attenuated neuronal damage. Controls included saline-injected and Sbt-only groups. The study provides robust *in vivo* evidence of Sbt’s antiepileptic potential, likely mediated through antioxidant and anti-inflammatory mechanisms. The study by Diandong et al. (2016) examines the immunomodulatory effects of SBT oil in a chronic stress rat model induced by restraint and exhaustive swimming. Doses of 5 and 10 mL/kg were administered *via* gavage for 21 days. SBT oil reversed stress-induced suppression of NK cell cytotoxicity and quantity, upregulated perforin and granzyme B expression, and modulated neuroendocrine-immune markers (e.g., cortisol, ACTH, 5-HT, IFN-γ). The study includes positive (soybean oil) and negative (stress model) controls, providing clear dose-dependent effects and mechanistic insights into SBT oil’s anti-stress and immunoenhancing properties. The study by Lan et al. (2023) explores the effects of *H. rhamnoides* polysaccharide (SBP) on HFD-induced cognitive dysfunction in mice. SBP was supplemented at 0.1% (w/w) in the diet for 12 weeks. It ameliorated neuroinflammation, synaptic dysfunction, gut barrier integrity, and microbiota dysbiosis. The study utilized behavioral tests, molecular biology, and microbiome analysis, with clear control groups (RC and HFD). SBP’s mechanism involves NF-κB suppression, CREB/BDNF/TrkB activation, and gut-brain axis modulation, highlighting its potential as a prebiotic neuroprotective agent.

While these studies provide valuable evidence for the neuroprotective and immunomodulatory effects of *H. rhamnoides* extracts, several limitations should be acknowledged. All three studies relied on crude extracts or polysaccharide fractions, which may contain multiple bioactive phytochemicals, raising the possibility of synergistic effects or PAINS risks, such as antioxidant-driven false positives in assay systems. The lack of phytochemical purification, dose-response curves, and *in vitro* validation limits mechanistic depth. Future research should focus on isolating active phytochemicals, conducting pharmacokinetic studies, and validating targets through genetic or pharmacological inhibition. Additionally, clinical trials are necessary to translate these findings into therapeutic applications, and more sophisticated models (e.g., human cell lines, organoids) should be employed to better understand the mechanisms of action.

### 5.4 Cutaneomucosal disorders

Cutaneomucosal disorders encompass pathophysiological conditions involving integumentary and mucosal epithelia, spanning infectious, inflammatory, allergic, and autoimmune etiologies. Prevalent manifestations comprise psoriasis vulgaris, eczematous dermatitis, atopic eczema, acneiform eruptions, and xerophthalmia. Such pathologies are manifested as cutaneous dysesthesia, pruritus, algia, erythematous reactions, and edematous responses, culminating in significant deterioration of health-related quality of life metrics.

Studies on vaginal inflammatory atrophy, skin hyperpigmentation, and microbial infections suggest (Solà Marsiñach and Cuenca, 2019) potential benefits of *H. rhamnoides* oil and its phytochemical palmitoleic acid (PA). In a clinical trial (*n* = 116 postmenopausal women), oral administration of 3 g/day of *H. rhamnoides* oil (∼24% PA) for 90 days improved the Vaginal Health Index without increasing serum estrogen, implying a distinct, possibly local anti-inflammatory mechanism. *In vitro*, PA inhibited key melanogenic enzymes (tyrosinase, TRP-2) and the transcription factor MITF in murine B16 melanoma cells, suggesting anti-melanogenic properties. Further *in vitro* assays demonstrated that PA (>1.0 mg/mL) prevented the adhesion of *Candida* albicans to human stratum corneum, and its calcium salt exhibited bactericidal activity against *Staphylococcus aureus* and Cutibacterium acnes, indicating a role in disrupting microbial adhesion and membrane integrity.

The evidence, while promising, is preliminary and exhibits significant limitations. The clinical study used a whole oil extract, making it impossible to attribute effects solely to PA due to potential synergies or antagonisms with other phytochemicals (e.g., sterols, tocopherols). The *in vitro* models (murine melanoma cells, microbial cultures) have limited translational relevance to human *in vivo* pathophysiology, and the effective concentrations of PA used, particularly in antimicrobial assays, may not be physiologically achievable or safe. The exact molecular mechanisms remain largely uncharacterized. Future research must prioritize using phylogenetically validated plant material, isolating PA to deconvolute its effects from the whole extract, and employing more human-relevant models (e.g., 3D tissue, animal disease models) with proper controls to establish dose-response relationships and definitive mechanisms. A major risk of PAINS is present, as fatty acids like PA can exhibit non-specific biological activity through surfactant-like effects, membrane disruption, or oxidation, which could account for the observed *in vitro* effects rather than specific target engagement. This must be rigorously ruled out in future mechanistic studies. Collectively, these observations establish a mechanistic rationale for Hippophae-derived fatty acids as targeted cutaneomucosal therapeutics.

### 5.5 Cardiovascular health management

Lifestyle modifications and demographic aging have established cardiovascular diseases as predominant global health burdens. Enhanced emphasis on cardiovascular health has promoted preventive strategies including dietary regulation, physical activity, tobacco abstinence, and alcohol restriction. Persistent unhealthy behaviors and hereditary influences maintain elevated cardiovascular disease incidence. Research confirms *H. rhamnoides* berries exhibit significant cardioprotective properties.

The study by Skalski et al. isolated (Skalski et al., 2021) six fractions (A–F: A, C, E as polyphenol-enriched; B, D, F as triterpenic acid-enriched) from *H. rhamnoides* fruits, leaves, and twigs. Antiplatelet activity was evaluated in a whole blood model after incubation for 30 min at concentrations of 5 and 50 μg/mL. Assessment was performed *via* flow cytometry (detecting P-selectin CD62P and activated GPIIb/IIIa PAC-1 expression) and the T-TAS thrombus analysis system. Results indicated that all fractions inhibited platelet activation to some extent, with the fruit polyphenol fraction (Fraction A) exhibiting the strongest activity. It significantly suppressed PAC-1 expression (in resting, ADP-, and collagen-activated platelets) and reduced the T-TAS area under the curve (AUC10), suggesting anticoagulant potential. Its mechanism was independent of the P2Y12 receptor (no change in VASP phosphorylation) and did not alter the platelet proteome. The study by Linderborg et al.(Linderborg et al., 2012) was a human postprandial crossover study. Healthy male volunteers consumed a yogurt meal containing 35 g of rapeseed oil and different *H. rhamnoides*/blackcurrant extraction residues (a dose equivalent to 400 g of fresh berries). Groups included: whole *H. rhamnoides*, CO_2_-extracted residue (rich in fiber and polyphenols), CO_2_+ethanol-extracted residue (rich in fiber, low in polyphenols), and a no-berry control meal. The primary outcome was the time course of plasma triacylglycerol (TAG) levels over 6 h postprandially. All berry meals delayed the time to peak postprandial lipemia but did not alter the total TAG incremental area under the curve (iAUC). The study suggested that this delaying effect was primarily attributable to the physical effects of dietary fiber (e.g., delayed gastric emptying, interference with lipid micellization), with polyphenols potentially playing a secondary role (as the effect was more sustained with the polyphenol-retaining fraction). This study by Stochmal et al. isolated (Stochmal et al., 2022) three flavonol phytochemicals from the polyphenol fraction of *H. rhamnoides* fruits: isorhamnetin (1), isorhamnetin-3-O-glucoside-7-O-rhamnoside (2), and isorhamnetin-3-O-glucoside-7-O-(3″-isovaleryl)-rhamnoside (3). Washed platelets and a whole blood model were used, with incubation for 30 min at 5 and 50 μg/mL. Evaluation was conducted using flow cytometry, T-TAS, and a colorimetric assay (collagen adhesion test). Compound 2 demonstrated the strongest overall activity, significantly inhibiting GPIIb/IIIa and P-selectin expression in collagen-activated platelets, and effectively reducing thrombus formation (AUC10) and platelet collagen adhesion. Its activity was superior to the parent phytochemical (1), suggesting that glycosylation and acylation modifications may enhance bioactivity. However, the precise molecular target(s) remain unidentified.

All three studies possess significant limitations. The concentrations used in the two *in vitro* studies (up to 50 μg/mL) raise questions regarding physiological relevance, and they lack detailed dose-response relationships (e.g., EC_50_/IC_50_ values) and in-depth mechanistic investigations into molecular targets. Although the whole blood model is superior to washed platelets, it still fails to fully replicate the complex *in vivo* vascular environment. The human study, while having higher clinical relevance, featured a small sample size, significant inter-individual variability, and was unable to dissect the specific contributions of fiber *versus* polyphenols, rendering its conclusions preliminary. Most critically, none of the studies adequately assessed the risk of PAINS. Phytochemicals of *H. rhamnoides*, such as polyphenols and fatty acids, are prone to oxidation, aggregation, or non-specific protein binding. The observed effects could represent false-positive outcomes rather than specific interactions with defined pharmacological targets. Future research must prioritize determining the *in vivo* effective concentrations of active phytochemicals and utilize knockout models or specific inhibitors to elucidate precise molecular mechanisms (e.g., identifying potential actions on classic antiplatelet targets like COX-1, P2Y12, or PAR1). Concurrently, rigorous PAINS filters should be applied, and structural optimization should be pursued to minimize non-specific effects. For complex mixtures like *H. rhamnoides* extracts, comparative activity assessments of isolated phytochemicals *versus* the whole extract are essential to distinguish the effects of single entities from synergistic interactions.

### 5.6 Antibacterial effects

Research demonstrates (Ma et al., 2023) that *H. rhamnoides* berry extracts and phytochemical phytochemicals (polyphenols, flavonoids, polysaccharides, volatile oils) exhibit antimicrobial properties. Ethanol extracts inhibit growth of *Bacillus* cereus, *Pseudomonas aeruginosa*, *Escherichia coli*, *S. aureus*, *Enterococcus* durans, and *Candida* albicans. n-Butanol fractions demonstrate antimicrobial activity toward *S. aureus*, *E. coli*, and *Bacillus subtilis*. Essential oils suppress the proliferation of *B. subtilis*, *Bacillus* thuringiensis, *Bacillus* cereus, *Bacillus* coagulans, *S. aureus*, and *E. coli*. Methanol extracts inhibit *Bacillus* cereus, *P. aeruginosa*, *S. aureus*, *Enterococcus faecalis*, Microsporum gypseum, and Trichophyton rubrum. Flavonoid fractions exert antimicrobial effects on *S. aureus*, *B. subtilis*, and *E. coli*. Polysaccharides display growth-inhibitory activity against *E. coli*, *B. subtilis*, *S. aureus*, and *Bacillus* licheniformis. Polyphenolic phytochemicals exhibit antimicrobial efficacy toward *E. coli*, *Salmonella typhi*, *Streptococcus* pneumoniae, and *S. aureus*.

The reported antimicrobial effects are almost exclusively based on *in vitro* agar diffusion or broth dilution assays. These assays are highly susceptible to interference from PAINS. Many polyphenols and flavonoids can cause false positives by precipitating proteins, oxidizing culture media components, or generating hydrogen peroxide. The MIC values are often quite high, questioning the physiological relevance. The lack of *in vivo* efficacy data in infection models significantly limits the interpretation of these findings. The antimicrobial potential may be more relevant for topical applications or food preservation than for systemic antibiotic therapy. Collectively, the extracts demonstrate potent antimicrobial efficacy *in vitro*, but robust scientific evidence for their pharmaceutical utilization requires confirmation in in vivo models.

### 5.7 Gastrointestinal disorders

*Hippophae rhamnoides* demonstrates therapeutic efficacy in the management and prevention of gastrointestinal pathologies. This study by Süleyman et al. evaluated (Süleyman et al., 2001) the anti-ulcer activity of a hexane extract (HRe-1) from the fruits of *H. rhamnoides* using two rat models: obligatory immobilization-induced (stress) and indomethacin-induced gastric ulcers. The extract was administered orally at a single dose of 1 mL/kg. In the stress model, HRe-1 was compared to the positive control drugs omeprazole (20 mg/kg) and famotidine (30 mg/kg); in the indomethacin model, it was compared to misoprostol (200 μg/kg). The key finding is that HRe-1 demonstrated significant protective effects in both models, outperforming the control drugs in most comparisons. In the stress model, the HRe-1 group showed significantly fewer ulcers and a smaller ulcer area (7.2, 10.1 mm^2^) than the control (15.6, 33.6 mm^2^), omeprazole (12.0, 22.0 mm^2^), and famotidine (9.0, 19.1 mm^2^) groups. The effect was even more pronounced in the indomethacin model, where only 4 out of 6 animals in the HRe-1 group developed ulcers, and the mean number and area of ulcers (1.3, 1.66 mm^2^) were drastically lower than those in the control (14.3, 31.0 mm^2^) and misoprostol (10.6, 26.33 mm^2^) groups. The authors propose that the mechanism is likely related to the synergistic antioxidant activity of numerous phytochemicals in the extract, such as vitamins C, E, and carotenoids, which may alleviate gastric mucosal damage by scavenging free radicals. However, this proposed mechanism remains speculative and was not experimentally verified. Ouyang et al. comprehensively investigates the protective effects of *H. rhamnoides* polysaccharide (SBP), which is extracted from the berries of *H. rhamnoides* (a species with well-documented ethnopharmacological use), on dextran sulfate sodium (DSS)-induced colitis in mice (Ouyang et al., 2024). The polysaccharide composition includes starch, cellulose, hemicelluloses, and pectin, though further structural characterization would enhance reproducibility. Using an *in vivo* murine model of acute colitis induced by 2.5% DSS administered orally for 7 days, the researchers evaluated a prophylactic SBP treatment regimen of 200 mg/kg body weight daily for 21 days, a dose selected based on prior hepatoprotective studies. Appropriate controls were employed, including a negative control group (CON, receiving sterile saline), a disease control group (DSS alone), and a treatment group (SBP + DSS), with additional validation through fecal microbiota transplantation (FMT) from SBP-treated mice. Ouyang et al. (2024) demonstrates that SBP significantly reduces inflammation (e.g., decreasing TNF-α, IL-6, IL-1β, and MPO), alleviates oxidative stress (e.g., increasing T-AOC, SOD, and CAT while reducing MDA), enhances intestinal barrier integrity (e.g., upregulating ZO-1, Occludin, Muc2, and CDX2), modulates gut microbiota (enriching Bifidobacterium and *Bacteroides* while reducing *Escherichia*), and increases production of short-chain fatty acids such as caproate, valerate, and propionate. These effects are robustly corroborated *via* fecal microbiota transplantation (FMT), confirming that the gut microbiota mediates SBP’s beneficial effects.

While both studies demonstrate promising gastroprotective effects of *H. rhamnoides* derivatives, several limitations and future directions must be considered: neither study fully characterized the bioactive composition of their extracts. Süleyman et al. (2001) used a hexane extract (HRe-1) rich in lipophilic phytochemicals but without quantification of key antioxidants, while Ouyang et al. (2024) administered a polysaccharide-rich fraction (SBP) lacking detailed structural profiling (e.g., monosaccharide sequence, molecular weight), which undermines reproducibility and mechanistic clarity. Both relied on prophylactic dosing in animal models, leaving therapeutic efficacy and human relevance unestablished. The proposed mechanisms, namely, antioxidant synergy in HRe-1 and microbiota modulation in SBP, remain speculative without targeted experimental validation (e.g., knockout models, antioxidant depletion, or human fecal cultures). Future studies should prioritize phytochemical standardization, dose-response relationships, pharmacokinetics, and validation in human-relevant models (e.g., organoids, humanized microbiota mice). Additionally, the risk of PAINS cannot be ruled out, particularly for the crude hexane extract, which may contain promiscuous activators or false positives in antioxidant assays. Clinical trials are essential to confirm efficacy and safety in humans.

### 5.8 Antioxidant effects

*Hippophae rhamnoides* demonstrates potent antioxidant capacity. This study by Geetha et al. utilized (Geetha et al., 2002) lymphocytes isolated from Sprague-Dawley rats to evaluate the antioxidant and immunomodulatory effects of 70% ethanolic extracts of seabuckthorn leaves and fruits. Chromium(VI) (10 μg/mL) was used to induce oxidative stress. The extracts were tested at a concentration of 500 μg/mL, which was identified as the minimal effective concentration for cytoprotection. Controls included untreated cells and chromium-only exposed cells. Key endpoints included cytotoxicity (LDH leakage), apoptosis (dual fluorescent staining), antioxidant enzymes (SOD, GPx), glutathione (GSH) levels, free radical production (DCFH-DA assay), nitric oxide, and lymphocyte proliferation (MTS assay with LPS/ConA mitogens). The study demonstrated that both leaf and fruit extracts significantly reduced chromium-induced oxidative damage and restored immune function, with leaf extract showing superior efficacy. The plant material was sourced from the Western Himalayas, India, and taxonomically identified as *H. rhamnoides*. This *in vivo* study investigated (Suleyman et al., 2002) the effects of *H. rhamnoides* fruit extract (HRe-1) and vitamin E on nicotine-induced oxidative stress in Sprague-Dawley rats. Nicotine was administered intraperitoneally at 0.5 mg/kg/day for 3 weeks. HRe-1 (1 mL/kg/day, intragastric) and vitamin E (75 mg/kg/day, intragastric) were used as interventions. Controls received vehicles only. Outcomes included erythrocyte MDA levels, antioxidant enzyme activities (SOD, GSH-Px, catalase), and plasma vitamin E and A levels (HPLC). HRe-1 effectively prevented nicotine-induced lipid peroxidation, restored SOD activity, enhanced GSH-Px activity, and increased plasma vitamin A levels. The extract was prepared from fruits collected in Erzurum, Turkey, and taxonomically verified.

Both studies provide valuable preliminary evidence of the antioxidant and cytoprotective properties of *H. rhamnoides*. However, they suffer from several limitations: The rich polyphenolic composition of *H. rhamnoides* extracts places them high on the list of potential PAINS. The observed effects in classic antioxidant assays (e.g., DCFH-DA for ROS, TBA for MDA) may be heavily confounded by non-biological, chemical artifacts. Polyphenols can directly quench radicals, chelate metal ions, reduce assay products, or exhibit autofluorescence, leading to false-positive results that masquerade as cellular antioxidant activity. The study by Geetha et al. is particularly vulnerable to these artifacts, as the effects seen in the cellular DCFH-DA assay could easily arise from direct chemical interference rather than true biological modulation of redox status. The use of single doses *in vitro* and *in vivo* limits dose-response characterization, and the lack of phytochemical standardization of the extracts undermines reproducibility. The *in vitro* model (rat lymphocytes) and *in vivo* model (nicotine-induced stress in rats) have limited translational relevance to human pathophysiology without further validation. Future studies should include dose-ranging experiments, detailed phytochemical profiling, use of human cell lines or clinical trials, and investigation of synergistic effects of bioactive phytochemicals. Overall, while promising, these findings require further rigorous and mechanistically detailed research to support therapeutic claims.

### 5.9 Anti-inflammatory effects

*Hippophae rhamnoides* flavonoids attenuate LPS-mediated body weight reduction and suppress macrophage, neutrophil, and total cell accumulation in bronchoalveolar lavage fluid, indicating therapeutic efficacy against chronic bronchitis. Research demonstrates (Ma et al., 2023) that *H. rhamnoides* total flavonoids possess moderate anti-inflammatory activity, inhibiting lipopolysaccharide (LPS)-induced interleukin-1β (IL-1β), interleukin-6 (IL-6), CXC chemokine ligand 1 (CXCL1), and mucin 5AC (MUC5AC) expression at transcriptional and translational levels in bronchial epithelial cells, while suppressing cyclooxygenase-2 (COX-2) expression and prostaglandin biosynthesis. Network pharmacology and molecular docking analyses suggest quercetin, isorhamnetin, and kaempferol exert anti-bronchitis effects *via* blockade of Fc epsilon RI, mitogen-activated protein kinase (MAPK), and vascular endothelial growth factor (VEGF) signaling cascades. *Hippophae* polysaccharides (Zhao et al., 2020) decrease toll-like receptor 4 (TLR4) and myeloid differentiation primary response 88 (MyD88) expression in lipopolysaccharide-activated intestinal epithelial cells and inhibit nuclear factor kappa B (NF-κB) phosphorylation, conferring protection against inflammatory damage. *Hippophae rhamnoides* methanol leaf extracts (Tanwar et al., 2018) inhibit inducible nitric oxide synthase (iNOS) and cyclooxygenase-2 (COX-2) expression while suppressing tumor necrosis factor-alpha (TNF-α) and interleukin-6 (IL-6) release in LPS-stimulated macrophages. *Hippophae* triterpenoids mitigate macrophage inflammation through nuclear factor kappa B (NF-κB), mitogen-activated protein kinase (MAPK), and nuclear factor erythroid 2-related factor 2 (Nrf2) pathways, suppressing LPS-induced inflammatory responses *in vitro*, indicating potential as natural anti-inflammatory nutraceutical agents.

Anti-inflammatory activity is a core feature of *H. rhamnoides* extracts. While the *in vitro* mechanistic data is extensive and often involves PAINS-prone phytochemicals, the consistency of the *in vivo* anti-inflammatory effects across different models (e.g., colitis, bronchitis, skin inflammation) provides strong corroborating evidence. The effects are likely due to a combination of direct (if weak) modulation of signaling pathways and indirect effects through antioxidant activity and microbiome interaction. Network pharmacology and molecular docking predictions require experimental validation and are highly speculative for PAINS phytochemicals. Collectively, *H. rhamnoides* and its bioactive phytochemicals demonstrate significant anti-inflammatory properties, offering valuable insights for novel anti-inflammatory agent development.

### 5.10 Others

Żuchowski also summarized (Żuchowski, 2023) recent evidence supporting the anti-obesity, radioprotective, and wound-healing effects of *H. rhamnoides* extracts, though noting that many studies remain preliminary and require further validation in physiologically relevant models. This study by Choi et al. evaluated (Choi et al., 2020) the anti-obesity effects of a combined extract (PHE) from *Patrinia scabiosaefolia* root (PS) and *H. rhamnoides* leaf (HR) using both *in vitro* and *in vivo* models. *In vitro*, 3T3-L1 preadipocytes were treated with PS, HR, or PHE (1:1 ratio) at concentrations ranging from 12.5 to 100 μg/mL. PHE showed synergistic inhibition of adipogenesis (*via* Oil Red O staining and Colby equation) and downregulated adipogenic genes (PPARγ, C/EBPα, FAS). For *in vivo* evaluation, male C57BL/6J mice were fed a high-fat diet (HFD) for 13 weeks to induce obesity, followed by 8 weeks of HFD supplemented with 0.25% or 0.5% PHE. Controls included normal diet and HFD-only groups. PHE significantly reduced body weight gain, adipose tissue size, hepatic lipid accumulation, and improved insulin sensitivity (OGTT). Serum triglycerides and cholesterol were also reduced. Plant materials were authenticated: PS roots from Kyungdong Market (Korea) and HR leaf powder (100% purity) from FromBio Co. Ltd. Extracts were prepared with 70% ethanol. The study demonstrates dose-dependent synergistic anti-obesity effects with robust *in vitro* and *in vivo* controls. This study by Goel et al. investigated (Goel et al., 2002) the radioprotective efficacy of RH-3, a 50% ethanolic extract of *H. rhamnoides* berries, against lethal whole-body irradiation in Swiss albino mice. The optimal radioprotective dose was determined to be 30 mg/kg (i.p.), administered 30 min before irradiation (10 Gy). Endpoints included 30-day survival, splenic CFU counts, hematological parameters (Hb, TLC, DLC), and *in vitro* free radical scavenging assays (OH^−^, O_2_^−^, lipid peroxidation). RH-3 significantly increased survival (81.7%), restored CFU counts, and improved recovery of hemoglobin and leukocyte counts. *In vitro*, RH-3 dose-dependently scavenged free radicals and inhibited lipid peroxidation. Berries were collected from Himachal Pradesh, India, and authenticated (voucher IHBT No. 1047). Controls included irradiated untreated mice and sham-treated groups. The study provides clear dose-response data and mechanistic insights into antioxidant and hematopoietic radioprotection. This study by Upadhyay et al. assessed (Upadhyay et al., 2009) the safety and efficacy of supercritical CO_2_-extracted *H. rhamnoides* seed oil (SBT) on burn wound healing in Sprague-Dawley rats. SBT was administered both orally (2.5 mL/kg) and topically (200 μL/wound) for 7 days. Silver sulfadiazine was used as a reference control. SBT significantly enhanced wound contraction, hydroxyproline, hexosamine, DNA, and protein content, upregulated MMP-2, MMP-9, collagen-III, and VEGF, and increased antioxidant markers (GSH, reduced ROS). Acute and sub-acute toxicity studies (up to 10 mL/kg p. o. for 28 days) showed no adverse effects. Seeds were collected from the Western Himalayas and authenticated (voucher SITS 20). The study combines robust wound healing assays with comprehensive safety profiling, including hematological, biochemical, and histopathological analyses.

The evidence base for these diverse applications is narrow and varies significantly in quality. The radioprotective effects, supported by *in vivo* models, are promising and mechanistically plausible, likely mediated through a combination of potent antioxidant scavenging of radiation-induced free radicals and anti-apoptotic mechanisms. In stark contrast, the claim for anti-obesity effects rests solely on a single *in vitro* adipogenesis assay. This model is highly susceptible to PAINS-related artifacts and nonspecific cytotoxicity, which can easily masquerade as inhibited differentiation. This finding requires robust validation in physiologically relevant *in vivo* dietary or genetic models of obesity before any conclusions can be drawn. The wound healing effects, particularly for burns, are supported by good *in vivo* evidence and represent one of the most translatable applications. The proposed mechanism, combining barrier repair (fatty acids), antioxidant activity (tocopherols, carotenoids), and anti-inflammatory action, is coherent and less prone to PAINS interference. However, optimization of formulation and demonstration of efficacy in human clinical trials are essential next steps.

## 6 Conclusions and perspectives

*Hippophae rhamnoides* represents a compelling model for translating ethnopharmacological wisdom into evidence-based applications, with its traditional multifunctional uses in Tibetan, Mongolian, Traditional Chinese, and its application in Eurasian medicine, strongly validated by the coherence between its phytochemical composition, pharmacological mechanisms, and clinical relevance, thus directly meets the core requirement for a clear biological rationale.

The plant’s efficacy in respiratory, digestive, cardiovascular, and inflammatory disorders stems from its diverse bioactive metabolites, which act synergistically to mediate conserved pathways. Specifically, flavonoids underpin its traditional respiratory and anti-inflammatory uses by blocking the NF-κB/MAPK signaling pathways to inhibit pro-inflammatory cytokine release; polysaccharides and fatty acids drive digestive health benefits through modulating gut microbiota (enriching Bifidobacterium and *Bacteroides*) and enhancing gastrointestinal barrier integrity, aligning with its traditional role in “invigorating the spleen and harmonizing the stomach”; triterpenoids (e.g., ursolic acid, oleanolic acid) and flavonoids collectively support cardiovascular function by regulating lipid metabolism and inhibiting platelet activation, validating its ethnomedical application of “promoting blood circulation to resolve stasis.” This structured alignment clarifies the molecular basis of *H. rhamnoides*’ ethnopharmacological heritage.

Despite these strengths, critical gaps remain: insufficient large-scale clinical trials to confirm preclinical findings in humans, incomplete characterization of synergistic interactions between bioactive phytochemical classes, and the need for standardized extracts to mitigate artifacts related to Pan-Assay Interference Compounds (PAINS). Future research should prioritize rigorous clinical validation of traditional formulations, mechanistic studies on phytochemical synergy, and sustainable exploitation of by-products (e.g., seed residues) within a circular bioeconomy framework.

In summary, *H. rhamnoides*’ ethnopharmacological relevance is robustly supported by its phytochemical-pharmacological correlations. By bridging traditional practice with modern science, while ensuring interpretive rigor through PAINS considerations, this work advances ethnopharmacological research and lays a solid foundation for the development of safe, effective phytotherapeutics derived from this versatile botanical resource.
