# Supplementary material for: Ethnopharmacology, phytochemistry, and pharmacology of sea buckthorn (Hippophae rhamnoides L.): a comprehensive review
Source: Front Pharmacol. 2026 Mar 6;17:1759697. doi: 10.3389/fphar.2026.1759697 (PMC13002580; doi:10.3389/fphar.2026.1759697)
Supplement: Supplementary file 1 [file Table1.pdf]

Supplementary Table 1. Phytochemicals of *H. rhamnoides* Berries

Classification	Phytochemical Designation	Structural Formula	Molecular Formula	Reference Citations
Flavonoids	quercetin-3-O-rutinoside	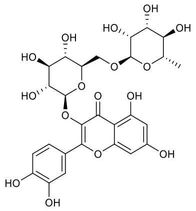	$C_{27}H_{30}O_{16}$	(Tkacz et al., 2019)
	isorhamnetin-3-O-rutinoside	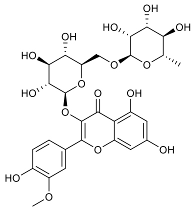	$C_{28}H_{32}O_{16}$	(Tkacz et al., 2019)
	Catechin	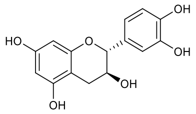	$C_{15}H_{14}O_6$	(Tkacz et al., 2019)
	Quercetin	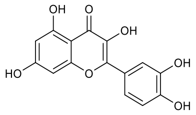	$C_{15}H_{10}O_7$	(Tkacz et al., 2019)
	Flavonoid	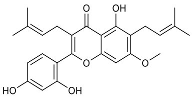	$C_{18}H_{16}O_8$	(Guo et al., 2017)
	Isorhamnetin	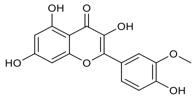	$C_{16}H_{12}O_7$	(Liu et al., 2021)
	Kaempferol	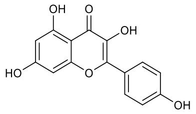	$C_{15}H_{10}O_6$	(Liu et al., 2021)
	Quercetin-3-O-glucoside	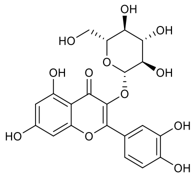	$C_{21}H_{20}O_{12}$	(Liu et al., 2021)
	Isorhamnetin-3-O-glucoside	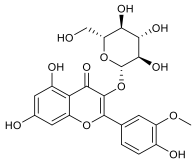	$C_{22}H_{22}O_{12}$	(Liu et al., 2021)
	Kaempferol-3-O-sophoroside-7-O-rhamnoside	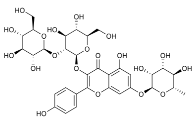	$C_{33}H_{40}O_{21}$	(Liu et al., 2021)
Phenolic Acids	Phenolic acid	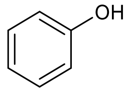		(Tkacz et al., 2019)
	Ferulic acid	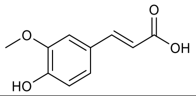	$C_{10}H_{10}O_4$	(Tkacz et al., 2019)

	Gallic acid	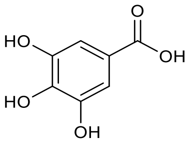	$C_7H_6O_5$	(Tkacz et al., 2019)
	Vanillic acid	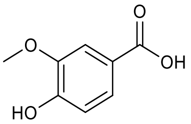	$C_8H_8O_4$	(Tkacz et al., 2019)
	Hydroxycaffeic			(Tkacz et al., 2019)
	Syringic	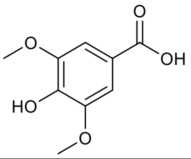	$C_9H_{10}O_5$	(Tkacz et al., 2019)
Vitamin	Vitamin B6	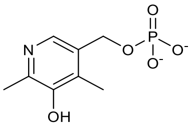	$C_8H_{10}NO_5P$--	(Stobdan et al., 2010)
	Niacin	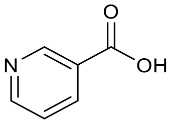	$C_6H_5NO_2$	(Stobdan et al., 2010)
	Vitamin A	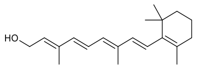	$C_{20}H_{30}O$	(Stobdan et al., 2010)
	Pantothenic acid	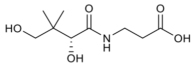	$C_9H_{17}NO_5$	(Stobdan et al., 2010)
Fatty acid	Myristic	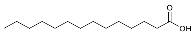	$C_{14}H_{28}O_2$	(Vaitkeviciene et al., 2019)
	Pentadecanoic	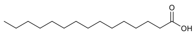	$C_{15}H_{30}O_2$	(Vaitkeviciene et al., 2019)
	Palmitic	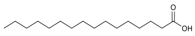	$C_{16}H_{32}O_2$	(Vaitkeviciene et al., 2019)
	Margaric	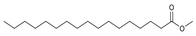	$C_{17}H_{34}O_2$	(Vaitkeviciene et al., 2019)
	Stearic	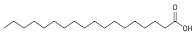	$C_{18}H_{36}O_2$	(Vaitkeviciene et al., 2019)
	Arachidic	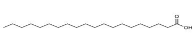	$C_{20}H_{40}O_2$	(Vaitkeviciene et al., 2019)
	Henicosanoic	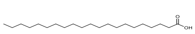	$C_{21}H_{42}O_2$	(Vaitkeviciene et al., 2019)
	Behenic	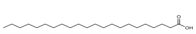	$C_{22}H_{44}O_2$	(Vaitkeviciene et al., 2019)
	Lignoceric	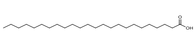	$C_{24}H_{48}O_2$	(Vaitkeviciene et al., 2019)
	Myristoleic	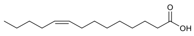	$C_{14}H_{26}O_2$	(Vaitkeviciene et al., 2019)
	Pentadecenoate	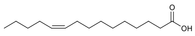		(Vaitkeviciene et al., 2019)

	Palmitoleic	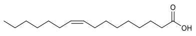	$C_{16}H_{30}O_2$	(Vaitkeviciene et al., 2019)
	Hexadecenoic	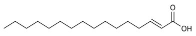	$C_{16}H_{30}O_2$	(Vaitkeviciene et al., 2019)
	Margaroleic	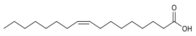		(Vaitkeviciene et al., 2019)
	Olei acid	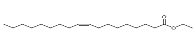	$C_{20}H_{38}O_2$	(Vaitkeviciene et al., 2019)
	cis-Vaccenic	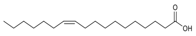	$C_{17}H_{32}O_2$	(Vaitkeviciene et al., 2019)
	Eicosenoic	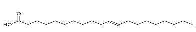	$C_{20}H_{38}O_2$	(Vaitkeviciene et al., 2019)
	Erucic	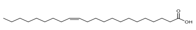	$C_{22}H_{42}O_2$	(Vaitkeviciene et al., 2019)
	Nervonic	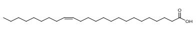	$C_{24}H_{46}O_2$	(Vaitkeviciene et al., 2019)
	Linoleic	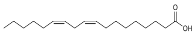	$C_{18}H_{32}O_2$	(Vaitkeviciene et al., 2019)
	γ-Linolenic	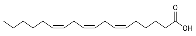	$C_{18}H_{30}O_2$	(Vaitkeviciene et al., 2019)
	α-Linolenic	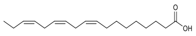	$C_{18}H_{30}O_2$	(Vaitkeviciene et al., 2019)
	Docosatetraenoic	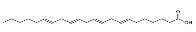	$C_{22}H_{36}O_2$	(Vaitkeviciene et al., 2019)
	Docosapentaensyre			(Vaitkeviciene et al., 2019)
Carotene	Lutein	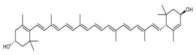	$C_{40}H_{56}O_2$	(Teleszko et al., 2015)
	Zeaxanthin	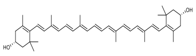	$C_{40}H_{56}O_2$	(Teleszko et al., 2015)
	β-Cryptoxanthin	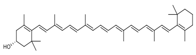	$C_{40}H_{56}O$	(Teleszko et al., 2015)
	δ-Carotene	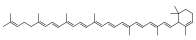	$C_{40}H_{56}$	(Teleszko et al., 2015)
	α-Carotene	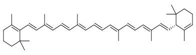	$C_{40}H_{56}$	(Teleszko et al., 2015)
	γ-Carotene	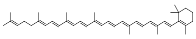	$C_{40}H_{56}$	(Teleszko et al., 2015)
	Cis β-Carotene	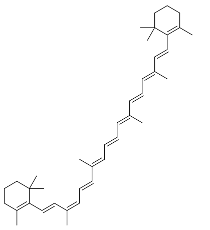	$C_{40}H_{56}$	(Teleszko et al., 2015)
	β-Carotene	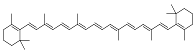	$C_{40}H_{56}$	(Teleszko et al., 2015)
	Lycopene	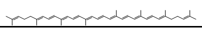	$C_{40}H_{56}$	(Tkacz et al.,

				2019)
Organic acid	L-malic acid	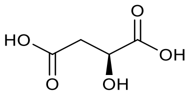	$C_4H_6O_5$	(Bal et al., 2011)
	D-malic acid	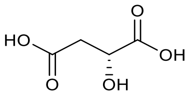	$C_4H_6O_5$	(Bal et al., 2011)
	Succinic acid	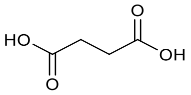	$C_7H_6O_4$	(Bal et al., 2011)
	Citric acid	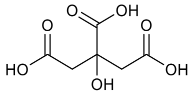	$C_6H_8O_7$	(Bal et al., 2011)
	Tartaric acid	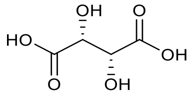	$C_4H_6O_6$	(Bal et al., 2011)
	Quinonic acid	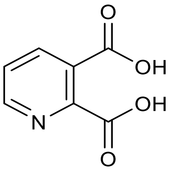		(Bal et al., 2011)
	Pyruvic acid	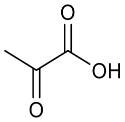	$C_3H_4O_3$	(Bal et al., 2011)
	Acetic acid	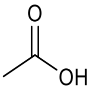	$C_2H_4O_2$	(Bal et al., 2011)
	Formic acid	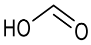	$C_{16}H_{17}N_3O_2$	(Bal et al., 2011)
	Oxalic acid	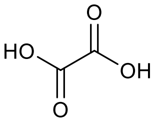	$C_2H_2O_4$	(Bal et al., 2011)
	Isocitric acid	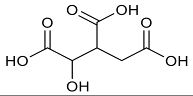	$C_6H_8O_7$	(Bal et al., 2011)
Amino acid	Aspartic acid	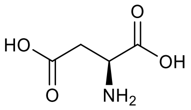	$C_4H_7NO_4$	(Bal et al., 2011)
	Serine	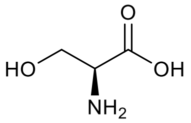	$C_{30}H_{48}O_6$	(Bal et al., 2011)
	Glutamic acid	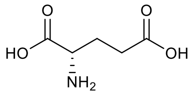	$C_5H_9NO_4$	(Bal et al., 2011)
	Glycine	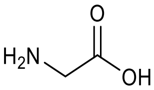	$C_2H_5CoNO_2$	(Bal et al., 2011)

Alanine	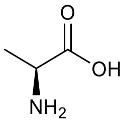	$C_3H_7NO_2$	(Bal et al., 2011)
Cysteine	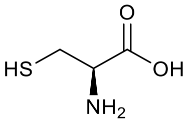	$C_3H_7NO_2S$	(Bal et al., 2011)
Tyrosine	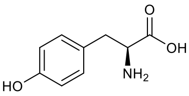	$C_9H_{11}NO_3$	(Bal et al., 2011)
Histidine	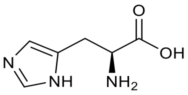	$C_6H_9N_3O_2$	(Bal et al., 2011)
Arginine	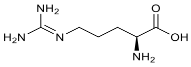	$C_6H_{13}N_3O_3$	(Bal et al., 2011)
Proline	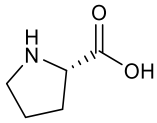	$C_5H_9NO_2$	(Bal et al., 2011)
Threonine	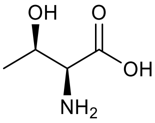	$C_4H_9NO_3$	(Bal et al., 2011)
Valine	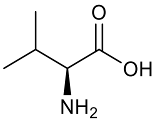	$C_5H_{11}NO_2$	(Bal et al., 2011)
Methionine	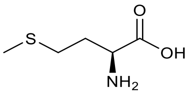	$C_5H_{11}NO_2S$	(Bal et al., 2011)
Isoleucine	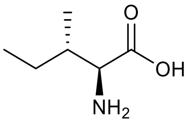	$C_6H_3D_{10}NO_2$	(Bal et al., 2011)
Leucine	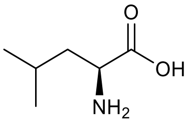		(Bal et al., 2011)
Phenylalanine	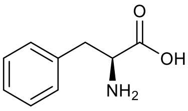	$C_9H_{11}NO_2$	(Bal et al., 2011)
Lysine	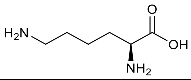	$C_6H_{14}N_2O_2$	(Bal et al., 2011)

Reference

Bal, L.M., Meda, V., Naik, S., and Satya, S.J.F.r.i. (2011). Sea buckthorn berries: A potential source of valuable nutrients for nutraceuticals and cosmoceuticals. *Food Res Int* 44(7)**,** 1718-1727. doi: 10.1016/j.foodres.2011.03.002.

Guo, R., Guo, X., Li, T., Fu, X., and Liu, R.H. (2017). Comparative assessment of phytochemical profiles, antioxidant and antiproliferative activities of Sea buckthorn

(Hippophaë rhamnoides L.) berries. *Food Chem* 221, 997-1003. doi: 10.1016/j.foodchem.2016.11.063.

Liu, S., Xiao, P., Kuang, Y., Hao, J., Huang, T., and Liu, E. (2021). Flavonoids from sea buckthorn: A review on phytochemistry, pharmacokinetics and role in metabolic diseases. *J Food Biochem* 45(5)**,** e13724. doi: 10.1111/jfbc.13724.

Stobdan, T., Chaurasia, O.P., Korekar, G., Mundra, S., Ali, Z., Yadav, A., et al. (2010). Attributes of seabuckthorn (Hippophae rhamnoides L.) to meet nutritional requirements in high altitude. *Defence Sci J* 60(2)**,** 226-230. doi: 10.14429/dsj.60.344.

Teleszko, M., Wojdyło, A., Rudzińska, M., Oszmiański, J., and Golis, T. (2015). Analysis of Lipophilic and Hydrophilic Bioactive Compounds Content in Sea Buckthorn (Hippophaë rhamnoides L.) Berries. *J Agric Food Chem* 63(16)**,** 4120-4129. doi: 10.1021/acs.jafc.5b00564.

Tkacz, K., Wojdyło, A., Turkiewicz, I.P., Bobak, Ł., and Nowicka, P. (2019). Anti-Oxidant and Anti-Enzymatic Activities of Sea Buckthorn (Hippophaë rhamnoides L.) Fruits Modulated by Chemical Components. *Antioxidants (Basel)* 8(12)**,** 618. doi: 10.3390/antiox8120618.

Vaitkeviciene, N., Jariene, E., Danilcenko, H., Kulaitiene, J., Mazeika, R., Hallmann, E., et al. (2019). Comparison of mineral and fatty acid composition of wild and cultivated sea buckthorn berries from Lithuania. *J Elem* 24(3)**,** 1101-1113. doi: 10.5601/jelem.2019.24.1.1759.
