# Supplementary material for: Ethnopharmacology, phytochemistry, and pharmacology of sea buckthorn (Hippophae rhamnoides L.): a comprehensive review
Source: Front Pharmacol. 2026 Mar 6;17:1759697. doi: 10.3389/fphar.2026.1759697 (PMC13002580; doi:10.3389/fphar.2026.1759697)
Supplement: Supplementary file 2 [file Table2.pdf]

Supplementary Table 2. Phytochemicals of *H. rhamnoides* Seeds

Classification	Phytochemical Designation	Structural Formula	Molecular Formula	Reference Citations
Flavonoids	Kaempferol–rhamnoside–glucoside	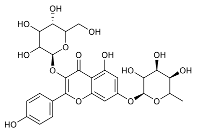		(Zhang et al., 2018)
	Kaempferol–pentoside	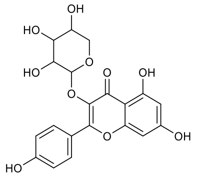		(Zhang et al., 2018)
	Kaempferol–hexoside	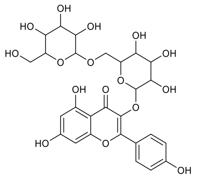		(Zhang et al., 2018)
	Kaempferol-3-O-rutinoside	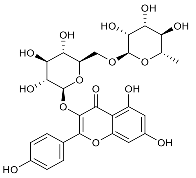	$C_{27}H_{30}O_{15}$	(Zhang et al., 2018)
	Kaempferol	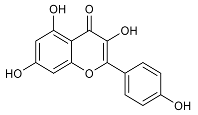	$C_{15}H_{10}O_6$	(Zhang et al., 2018)
	Quercetin-3-O-(6”-benzoyl)-β-galactoside			(Danielski and Shahidi, 2024)
	Quercetin sulfate	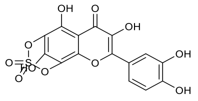		(Danielski and Shahidi, 2024)
	Mearnsetin diglucoside	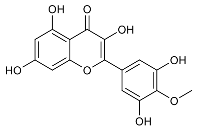		(Danielski and Shahidi, 2024)
	Gallocatechin	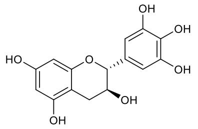	$C_{15}H_{14}O_7$	(Danielski and Shahidi, 2024)
	Catechin-O-dihexoside			(Danielski and Shahidi, 2024)

	Apigenin	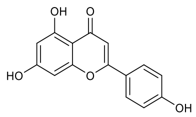	$C_{15}H_{10}O_5$	(Danielski and Shahidi, 2024)
	(+)-Catechin	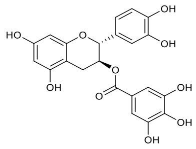	$C_{15}H_{14}O_6$	(Danielski and Shahidi, 2024)
	Rutin	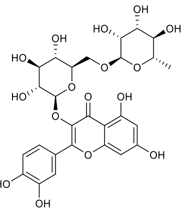	$C_{27}H_{30}O_{16}$	(Zhang et al., 2018)
	Quercetin-7-O-rutinoside	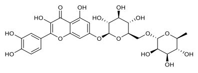	$C_{27}H_{30}O_{16}$	(Zhang et al., 2018)
	Quercetin-3-O-glucoside-7-O-rhamnoside	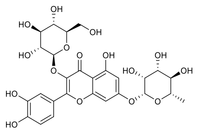	$C_{27}H_{30}O_{16}$	(Zhang et al., 2018)
	Quercetin-3-O-glucoside	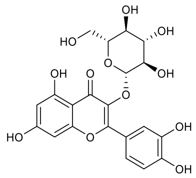	$C_{21}H_{20}O_{12}$	(Zhang et al., 2018)
	Quercetin	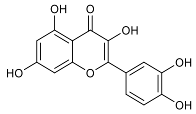	$C_{15}H_{10}O_7$	(Zhang et al., 2018)
	Gallocatechin	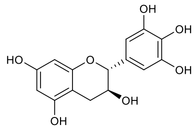	$C_{15}H_{14}O_7$	(Zhang et al., 2018)
	Epigallocatechin	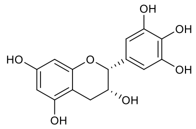	$C_{15}H_{14}O_7$	(Zhang et al., 2018)
	Catechin	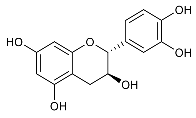	$C_{15}H_{14}O_6$	(Zhang et al., 2018)
	Myricetin	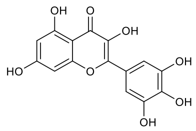	$C_{15}H_{10}O_8$	(Zhang et al., 2018)

	Isorhamnetin-3-O-rutinoside	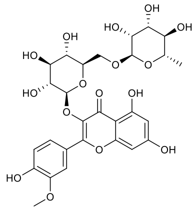		(Zhang et al., 2018)
	Isorhamnetin-3-O-glucoside-7-O-rhamnoside	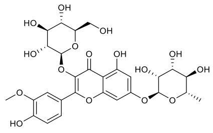	$C_{25}H_{48}O_4$	(Zhang et al., 2018)
	Isorhamnetin-3-O-glucoside	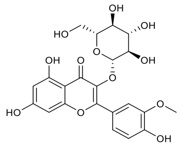	$C_{22}H_{22}O_{12}$	(Zhang et al., 2018)
	Isorhamnetin	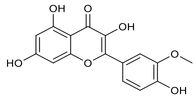	$C_{16}H_{12}O_7$	(Zhang et al., 2018)
Phenol Phytochemical	Ellagic derivative IV			(Danielski and Shahidi, 2024)
	Ellagic derivative II			(Danielski and Shahidi, 2024)
	Ellagic acid pentoside	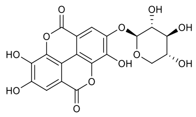		(Danielski and Shahidi, 2024)
	3,4,8,9,10-Pentahydroxy dibenzo [b,d]pyran-6-one	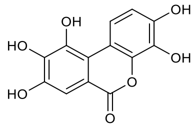		(Danielski and Shahidi, 2024)
Fatty acids	stearic acid	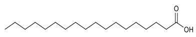	$C_{18}H_{36}O_2$	(Dulf, 2012)
	palmitic acid	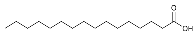	$C_{16}H_{32}O_2$	(Dulf, 2012)
	myristic acid	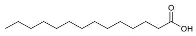	$C_{14}H_{28}O_2$	(Dulf, 2012)
	margaric acid	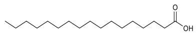	$C_{17}H_{34}O_2$	(Dulf, 2012)
	arachidic acid	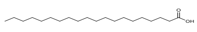	$C_{20}H_{40}O_2$	(Dulf, 2012)
	linolenic acid	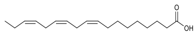	$C_{18}H_{30}O_2$	(Dulf, 2012)
	linoleic acid	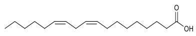	$C_{18}H_{32}O_2$	(Dulf, 2012)
	Tri-O-caffeoylshikimic acid			(Danielski and Shahidi, 2024)
	trans-Cinnamic acid	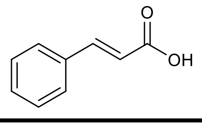	$C_9H_8O_2$	(Danielski and Shahidi, 2024)

	Protocatechuic acid	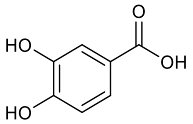	$C_7H_6O_4$	(Danielski and Shahidi, 2024)
	p-Hydroxybenzoic acid	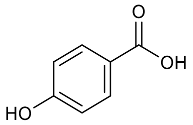	$C_7H_6O_3$	(Danielski and Shahidi, 2024)
	p-Coumaroyl malonyldihexoside			(Danielski and Shahidi, 2024)
	p-Coumaric acid	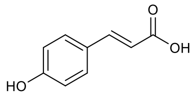	$C_9H_8O_3$	(Danielski and Shahidi, 2024)
	O-Methylgallic acid	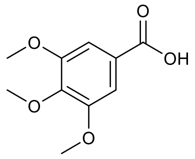		(Danielski and Shahidi, 2024)
	Hydroxygallic acid	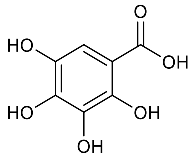		(Danielski and Shahidi, 2024)
	Hydroxycaffeic acid	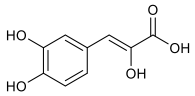		(Danielski and Shahidi, 2024)
	Ferulic acid	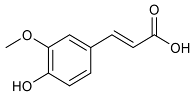	$C_{10}H_{10}O_4$	(Danielski and Shahidi, 2024)
	Caffeoylquinate shikimate derivative	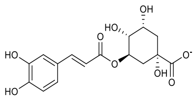		(Danielski and Shahidi, 2024)
	Caffeic acid	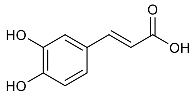	$C_9H_8O_4$	(Danielski and Shahidi, 2024)
	oleic acid	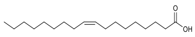	$C_{18}H_{34}O_2$	(Dulf, 2012)
	eicosenoic acid	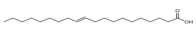	$C_{20}H_{38}O_2$	(Dulf, 2012)
	cis-vaccenic acid	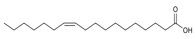	$C_{18}H_{34}O_2$	(Dulf, 2012)
	palmitoleic acid	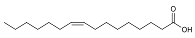	$C_{16}H_{30}O_2$	(Dulf, 2012)

Organic acids	Quinic acid pentoside	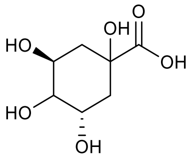		(Zhang et al., 2018)
	Cinnamyl pentoside	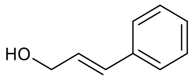		(Zhang et al., 2018)
	Chlorogenic acid	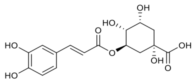	$C_{16}H_{18}O_9$	(Zhang et al., 2018)
	Quinic acid	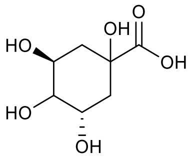	$C_7H_{12}O_6$	(Zhang et al., 2018)
	Protocatechuic acid	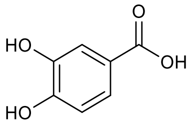	$C_7H_6O_4$	(Zhang et al., 2018)
	Dihydrocinnamyl pentoside	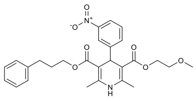		(Zhang et al., 2018)

Reference

Danielski, R., and Shahidi, F. (2024). Phenolic composition and bioactivities of sea buckthorn (Hippophae rhamnoides L.) fruit and seeds: an unconventional source of natural antioxidants in North America. *J Sci Food Agric* 104(9), 5553-5564. doi: 10.1002/jsfa.13386.

Dulf, F.V. (2012). Fatty acids in berry lipids of six sea buckthorn (Hippophae rhamnoides L., subspecies carpatica) cultivars grown in Romania. *Chem Cent J* 6(1), 106. doi: 10.1186/1752-153x-6-106.

Zhang, J., Wang, C., Wang, C., Sun, B., and Qi, C. (2018). Understanding the role of extracts from sea buckthorn seed residues in anti-melanogenesis properties on B16F10 melanoma cells. *Food Funct* 9(10), 5402-5416. doi: 10.1039/c8fo01427b.
