# Supplementary material for: Ethnopharmacology, phytochemistry, and pharmacology of sea buckthorn (Hippophae rhamnoides L.): a comprehensive review
Source: Front Pharmacol. 2026 Mar 6;17:1759697. doi: 10.3389/fphar.2026.1759697 (PMC13002580; doi:10.3389/fphar.2026.1759697)
Supplement: Supplementary file 3 [file Table3.pdf]

Supplementary Table 3. Phytochemicals of *Hippophae* Foliage

**Classification**	**Phytochemical Designation**	**Structural Formula**	**Molecular Formula**	**Reference Citations**
Flavonoids	Kaempferol	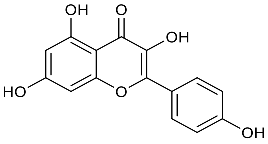	$C_{15}H_{10}O_6$	(Ding et al., 2023)
	Kaempferol-3-O-glucoside	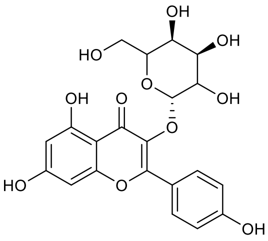	$C_{21}H_{20}O_{11}$	(Ding et al., 2023)
	Kaempferol-3-O-rutinoside	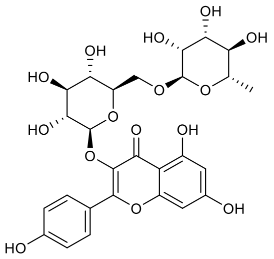	$C_{27}H_{30}O_{15}$	(Ding et al., 2023)
	Kaempferol-3-O-β-D-(6″-O-p-coumaryl) glyco side	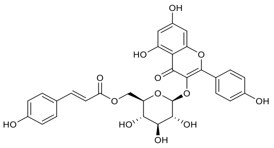	$C_{30}H_{26}O_{13}$	(Ding et al., 2023)
	Kaempferol-3-O-glucoside-7-O-rhamnoside	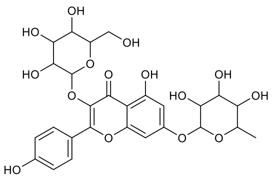	$C_{27}H_{30}O_{15}$	(Ding et al., 2023)
	Kaempferol-3-O-sophoroside-7-O-rhamnoside	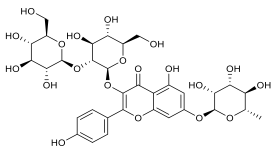	$C_{33}H_{40}O_{20}$	(Ding et al., 2023)
	Quercetin	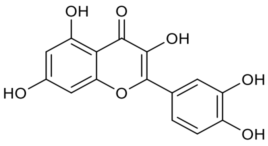	$C_{15}H_{10}O_7$	(Ding et al., 2023)
	Quercetin-3-O-glucoside	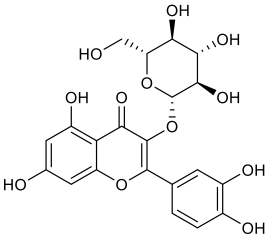	$C_{21}H_{20}O_{12}$	(Ding et al., 2023)

	Quercetin-3-O-galactoside	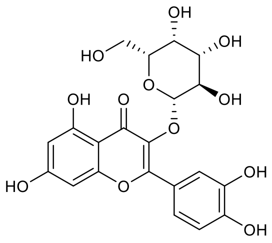	$C_{21}H_{20}O_{12}$	(Ding et al., 2023)
	Rutin	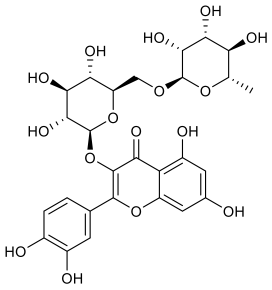	$C_{27}H_{30}O_{16}$	(Ding et al., 2023)
	Rhamnin	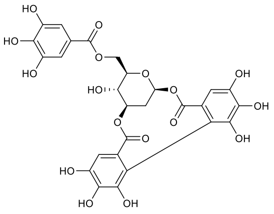	$C_{27}H_{22}O_{18}$	(Ding et al., 2023)
	Narcissin	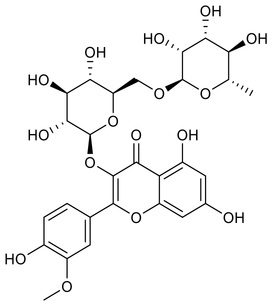	$C_{28}H_{32}O_{16}$	(Yuca et al., 2022)
	Quercetin-7-O-rhamnoside	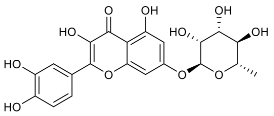	$C_{21}H_{20}O_{11}$	(Ding et al., 2023)
	Quercetin-3-O-glucoside-7-O-rhamnoside	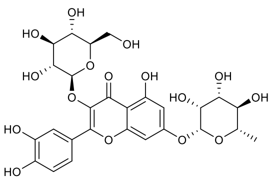	$C_{27}H_{30}O_{16}$	(Ding et al., 2023)
	Quercetin-3，7-O-diglucoside	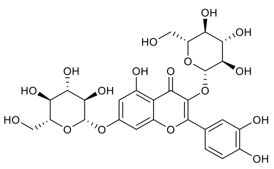	$C_{27}H_{30}O_{17}$	(Ding et al., 2023)
	Isorhamnetin	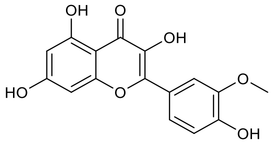	$C_{16}H_{12}O_7$	(Ding et al., 2023)

	Isorhamnetin-3-O-glucoside	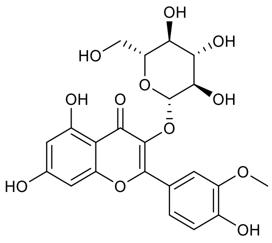	$C_{22}H_{22}O_{12}$	(Ding et al., 2023)
	Isorhamnetin-3-O-rutinoside	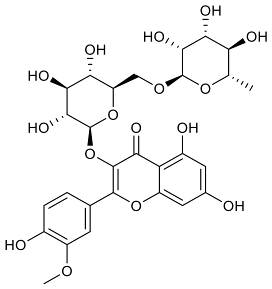	$C_{28}H_{32}O_{16}$	(Ding et al., 2023)
	Isorhamnetin-7-O-rhamnoside	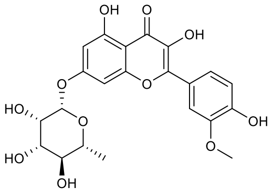	$C_{22}H_{22}O_{11}$	(Ding et al., 2023)
	Isorhamnetin-3-O-rutinoside-7-O-rhamnoside	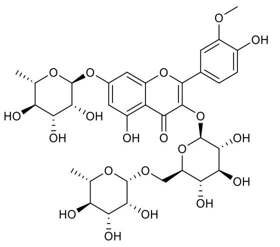	$C_{34}H_{42}O_{20}$	(Ding et al., 2023)
	Isorhamnetin-3-O-glucoside-7-O-rhamnoside	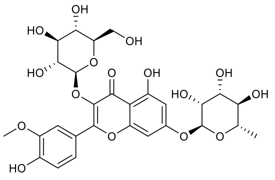	$C_{28}H_{32}O_{16}$	(Ding et al., 2023)
	Isorhmnetin-3-O-galactose-7-O-rhamnoside	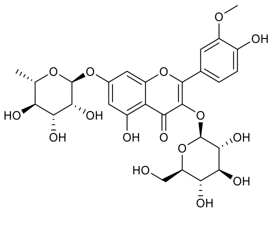	$C_{28}H_{32}O_{16}$	(Ding et al., 2023)
	Myricetin	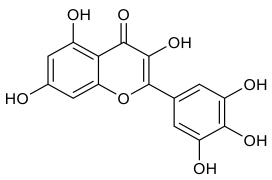	$C_{15}H_{10}O_8$	(Ding et al., 2023)
	Apigenin	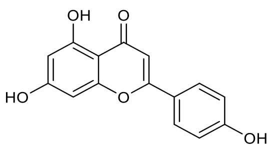	$C_{15}H_{10}O_5$	(Ding et al., 2023)

	4，4'-Dihydroxy-2'-methoxychalcone	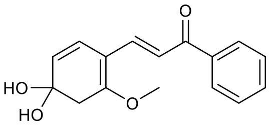	$C_{16}H_{14}O_4$	(Ding et al., 2023)
	Hippophaeoside A	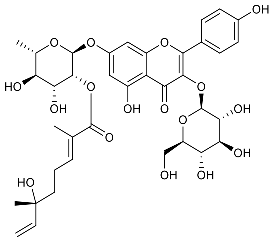	$C_{37}H_{44}O_{17}$	(Ding et al., 2023)
	Hippophaeoside B	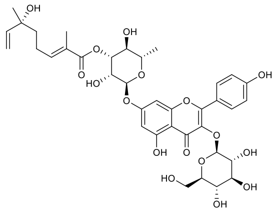	$C_{37}H_{44}O_{17}$	(Ding et al., 2023)
	Hippophaeoside C	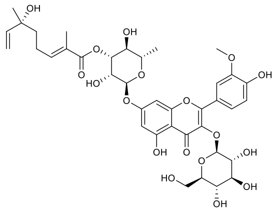	$C_{38}H_{46}O_{18}$	(Ding et al., 2023)
Phenolic Acids	Salicylic acid	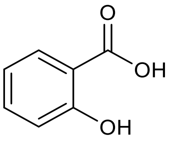	$C_7H_6O_3$	(Ding et al., 2023)
	Gentisic acid	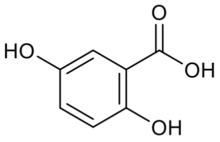	$C_7H_6O_4$	(Ding et al., 2023)
	Methyl gallate	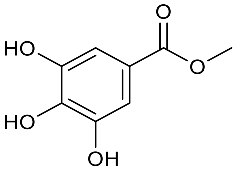	$C_8H_8O_5$	(Ding et al., 2023)
	p-Hydroxybenzoic acid	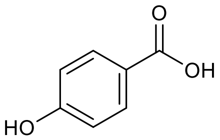	$C_7H_6O_3$	(Ding et al., 2023)
	Gallic acid	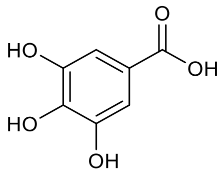	$C_7H_6O_5$	(Ding et al., 2023)

	Protocatechuic acid	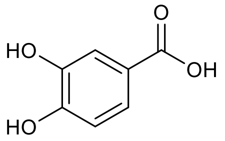	$C_7H_6O_4$	(Ding et al., 2023)
	Vanillic acid	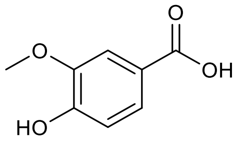	$C_8H_8O_4$	(Ding et al., 2023)
	Syringic acid	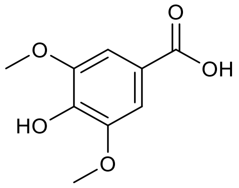	$C_9H_{10}O_5$	(Ding et al., 2023)
	(E) -Cinnamic acid	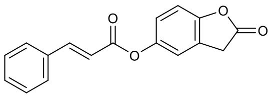	$C_9H_8O_2$	(Ding et al., 2023)
	Caffeic acid	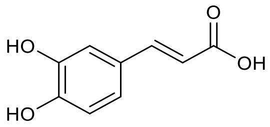	$C_9H_8O_4$	(Ding et al., 2023)
	Ferulic acid	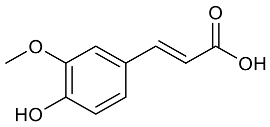	$C_{10}H_{10}O_4$	(Ding et al., 2023)
	Sinapic acid	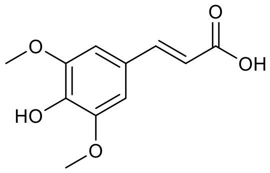	$C_{11}H_{12}O_5$	(Ding et al., 2023)
	1-Feruloyl-β-D-glucopyranoside	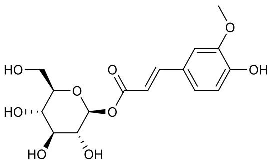	$C_{16}H_{20}O_9$	(Ding et al., 2023)
	Chlorogenic acid	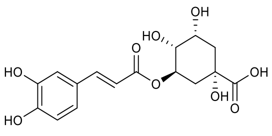	$C_{16}H_{18}O_9$	(Ding et al., 2023)
Polyphenols	Gossypol	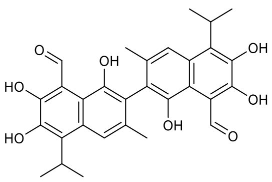	$C_{30}H_{30}O_8$	(Ding et al., 2023)

Hydrolysable Tannins	1，2，6-Tri-O-galloyl-β-D-glucopyranose	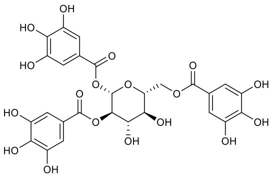	$C_{27}H_{24}O_{18}$	(Yang et al., 2019)
	1，3，6-Tri-O-galloyl-β-D-glucopyranose	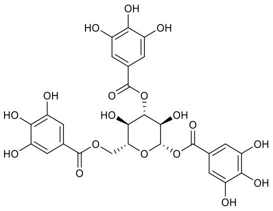	$C_{27}H_{24}O_{18}$	(Yang et al., 2019)
	1，4，6-Tri-O-galloyl-β-D-glucopyranose	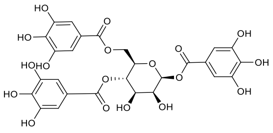	$C_{27}H_{24}O_{18}$	(Yang et al., 2019)
	1，2，3，6-Tetra-O-galloyl-β-D-glucopyranose	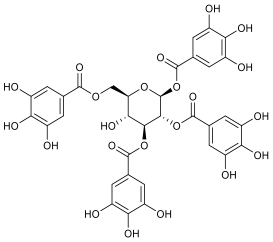	$C_{34}H_{28}O_{22}$	(Yang et al., 2019)
	1，3，4，6-Tetra-O-galloyl-β-D-glucopyranose	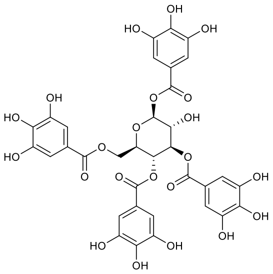	$C_{34}H_{28}O_{22}$	(Yang et al., 2019)
	1，2，3，4，6-Penta-O-galloyl-β-D-glucopyranose	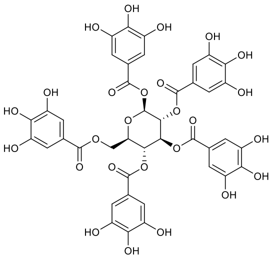	$C_{41}H_{32}O_{26}$	(Yang et al., 2019)
Ellagitannins	Isostrictinin	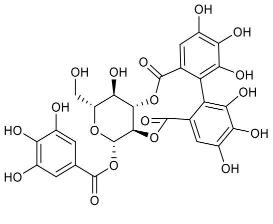	$C_{27}H_{22}O_{18}$	(Yang et al., 2019)

	Ellagic acid	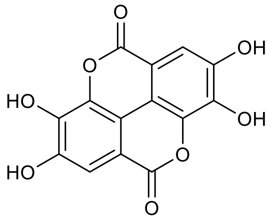	$C_{14}H_6O_8$	(Ding et al., 2023)
	Nobotanin D	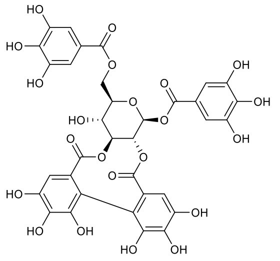	$C_{34}H_{26}O_{22}$	(Yang et al., 2019)
	Pterocaryanin C	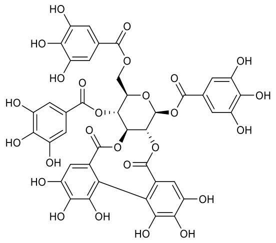	$C_{41}H_{30}O_{26}$	(Yang et al., 2019)
	Strictinin	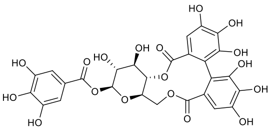	$C_{27}H_{22}O_{18}$	(Yang et al., 2019)
	Tellimagrandin I	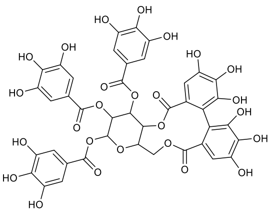	$C_{34}H_{26}O_{22}$	(Ding et al., 2023)
	Tellimagrandin II	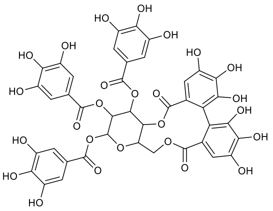	$C_{41}H_{30}O_{26}$	(Ding et al., 2023)
	Pedunculagin	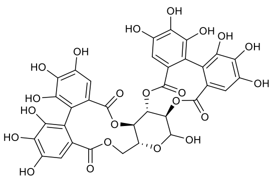	$C_{34}H_{24}O_{22}$	(Ding et al., 2023)

	Casuarictin	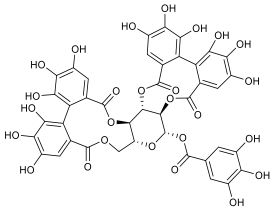	$C_{41}H_{28}O_{26}$	(Ding et al., 2023)
	Castalagin	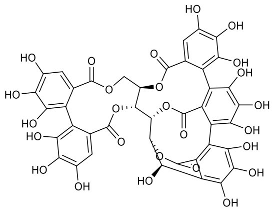	$C_{41}H_{26}O_{26}$	(Ding et al., 2023)
	Vescalagin	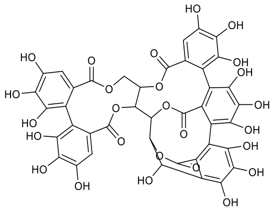	$C_{41}H_{26}O_{26}$	(Ding et al., 2023)
	Casuarinin	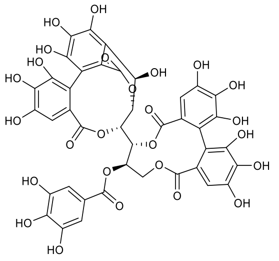	$C_{41}H_{28}O_{26}$	(Ding et al., 2023)
	Stachyurin	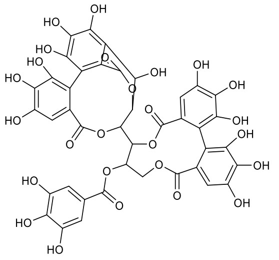	$C_{41}H_{28}O_{26}$	(Ding et al., 2023)
Terpenoids	Vomifoliol	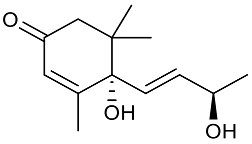	$C_{13}H_{20}O_3$	(Ding et al., 2023)
	α-Amyrin	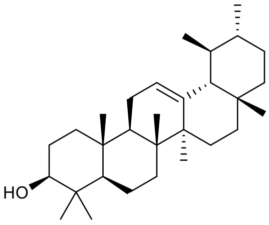	$C_{30}H_{50}O$	(Ding et al., 2023)

	β-Amyrin	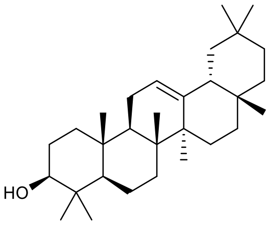	$C_{30}H_{50}O$	(Ding et al., 2023)
	Squalene	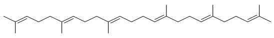	$C_{30}H_{50}O$	(Ding et al., 2023)
	Uvaol	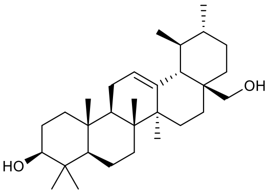	$C_{30}H_{50}O_2$	(Ding et al., 2023)
	Erythrodiol	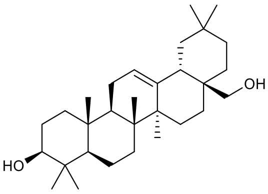	$C_{30}H_{50}O_2$	(Ding et al., 2023)
	Ursolic aldehyde	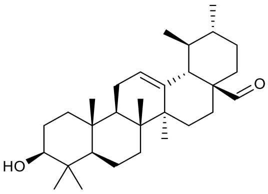	$C_{30}H_{48}O_2$	(Ding et al., 2023)
	Oleanolic aldehyde	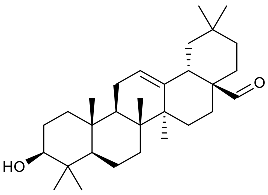	$C_{30}H_{48}O_2$	(Ding et al., 2023)
	Ursolic acid	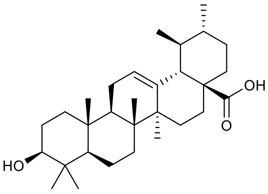	$C_{30}H_{48}O_3$	(Ding et al., 2023)
	2α-Hydroxyursolic acid	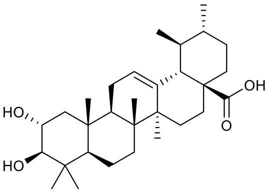	$C_{30}H_{48}O_4$	(Ding et al., 2023)
	23-Hydroxyursolic acid	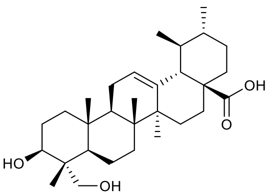	$C_{30}H_{48}O_4$	(Ding et al., 2023)

	Maslinic acid	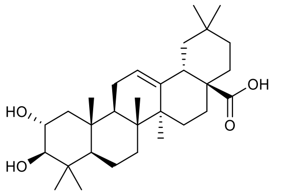	$C_{30}H_{48}O_4$	(Ding et al., 2023)
	Pomolic acid	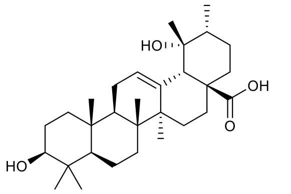	$C_{30}H_{48}O_4$	(Ding et al., 2023)
	28-Noroleana-12，17-dien-3β-ol	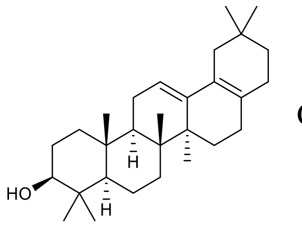	$C_{29}H_{46}O$	(Ding et al., 2023)
	Lupeol	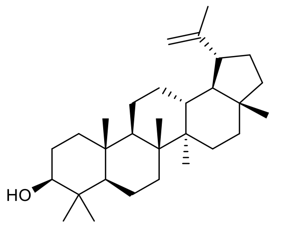	$C_{30}H_{50}O$	(Ding et al., 2023)
Steroids	Ergost-5-en-3β-ol	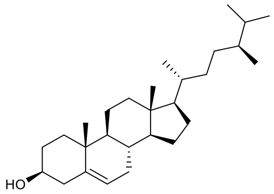	$C_{29}H_{50}O$	(Ding et al., 2023)
	β-Sitosterol	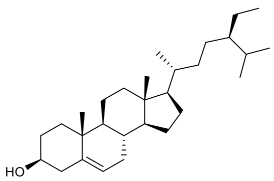	$C_{29}H_{50}O$	(Ding et al., 2023)
	Stigmast-7-en-3β-ol	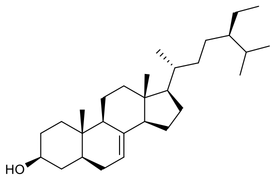	$C_{30}H_{50}O$	(Ding et al., 2023)
	Obtusifoliol	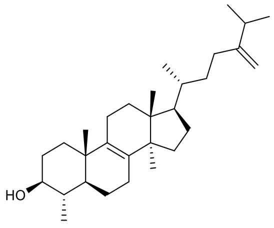	$C_{30}H_{50}O$	(Ding et al., 2023)

	Cycloartenol	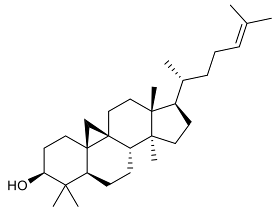	$C_{30}H_{50}O$	(Ding et al., 2023)
	24-Methylene cycloartenol	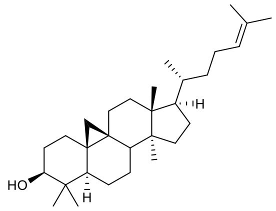	$C_{31}H_{52}O$	(Ding et al., 2023)
Inositol derivatives	Pinitol	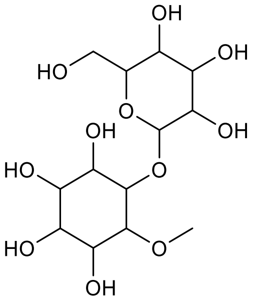	$C_7H_{14}O_6$	(Yang et al., 2019)
	ArjunglucosideI	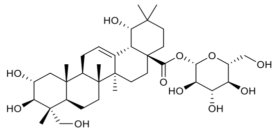	$C_{36}H_{58}O_{11}$	(Yuca et al., 2022)

Reference

Ding, Z., Ye, J., Ma, J., He, X., Wang, Z., Liang, L., et al. (2023). Research Progress in Chemical Constituents and Pharmacological Effects of Hippophae rhamnoides Leaves. *World Chinese Medicine* 18(5)**,** 714-720. doi: 10.3969 / j.issn.1673-7202.2023.05.023.

Yang, Z., Zheng, W., Zhang, K., Sun, L., Bao, F., Zhang, L., et al. (2019). Inhibitory effects of tannins from leaves of Hippophae rhamnoids and their anti-inflammatory and anti-obesity effects. *Chinese Traditional and Herbal Drugs* 50(12)**,** 2809-2816. doi: 10.7501/j.issn.0253-2670.2019.12.010.

Yuca, H., Özbek, H., Demirezer, L.Ö., Sevindik, H.G., Kazaz, C., and Güvenalp, Z. (2022). α-Glucosidase and α-amylase inhibitory potential of main compounds and drug candidates from Elaeagnus rhamnoides (L.) A. Nelson. *Chemical Papers* 76(2)**,** 913-922. doi: 10.1007/s11696-021-01904-4.
